# Single‐cell rapid identification, in situ viability and vitality profiling, and genome‐based source‐tracking for probiotics products

**DOI:** 10.1002/imt2.117

**Published:** 2023-05-25

**Authors:** Jia Zhang, Lihui Ren, Lei Zhang, Yanhai Gong, Teng Xu, Xiaohang Wang, Cheng Guo, Lei Zhai, Xuejian Yu, Ying Li, Pengfei Zhu, Rongze Chen, Xiaoyan Jing, Gongchao Jing, Shiqi Zhou, Mingyue Xu, Chen Wang, Changkai Niu, Yuanyuan Ge, Bo Ma, Gaishuang Shang, Yunlong Cui, Su Yao, Jian Xu

**Affiliations:** ^1^ Single‐Cell Center, CAS Key Laboratory of Biofuels, Shandong Key Laboratory of Energy Genetics, Qingdao Institute of Bioenergy and Bioprocess Technology, Chinese Academy of Sciences Qingdao Shandong China; ^2^ Shandong Energy Institute Qingdao Shandong China; ^3^ Qingdao New Energy Shandong Laboratory Qingdao Shandong China; ^4^ University of Chinese Academy of Sciences Beijing China; ^5^ College of Information Science & Engineering Ocean University of China Qingdao Shandong China; ^6^ Qingdao Branch of China United Network Communications Co., Ltd. Qingdao Shandong China; ^7^ Eastsea Pharma Co., Ltd. Qingdao Shandong China; ^8^ China National Research Institute of Food and Fermentation Industries Co., Ltd., China Center of Industrial Culture Collection Beijing China; ^9^ Qingdao Single‐Cell Biotech. Co., Ltd. Qingdao Shandong China

**Keywords:** identification, probiotics, quality assessment, Raman‐activated cell sorting, single‐cell sequencing, vitality

## Abstract

Rapid expansion of the probiotics industry demands fast, sensitive, comprehensive, and low‐cost strategies for quality assessment. Here, we introduce a culture‐free, one‐cell‐resolution, phenome‐genome‐combined strategy called Single‐Cell Identification, Viability and Vitality tests, and Source‐tracking (SCIVVS). For each cell directly extracted from the product, the fingerprint region of D_2_O‐probed single‐cell Raman spectrum (SCRS) enables species‐level identification with 93% accuracy, based on a reference SCRS database from 21 statutory probiotic species, whereas the C–D band accurately quantifies viability, metabolic vitality plus their intercellular heterogeneity. For source‐tracking, single‐cell Raman‐activated Cell Sorting and Sequencing can proceed, producing indexed, precisely one‐cell‐based genome assemblies that can reach ~99.40% genome‐wide coverage. Finally, we validated an integrated SCIVVS workflow with automated SCRS acquisition where the whole process except sequencing takes just 5 h. As it is >20‐fold faster, >10‐time cheaper, vitality‐revealing, heterogeneity‐resolving, and automation‐prone, SCIVVS is a new technological and data framework for quality assessment of live‐cell products.

## Highlights

- We introduce a culture‐free, one‐cell‐resolution, phenome‐genome‐combined strategy called Single‐Cell Identification, Viability and Vitality tests, and Source‐tracking (SCIVVS) for rapid quality assessment of probiotics products.
- SCIVVS enables species‐level identification with 93% accuracy and quantifies viability, metabolic vitality plus their intercellular heterogeneity.
- For source‐tracking a cell, SCIVVS can reach ~99.40% genome‐wide coverage for various *Lactobacillus*, *Bifidobacterium*, and *Streptococcus* spp.
- SCIVVS is >20‐fold faster, >10‐time cheaper, vitality‐revealing, heterogeneity‐resolving, and automation‐prone.
- SCIVVS is a new technological and data framework that can transform quality evaluation, biomanufacturing‐process monitoring, and intellectual‐property protection of probiotics and other live‐cell products.

## Graphical abstract

The rapid expansion of the probiotics industry demands fast, sensitive, comprehensive, and low‐cost methods for quality assessment of such live‐cell products. However current methods are slow, tedious, and incapable of quantifying in situ vitality or its heterogeneity. Here, we introduce a culture‐free, one‐cell‐resolution, phenome‐genome‐combined strategy called Single‐Cell Identification, Viability and Vitality tests, and Source‐tracking (SCIVVS). As it is >20‐fold faster, >10‐time cheaper, vitality‐revealing, heterogeneity‐resolving, and automation‐prone, SCIVVS is a new technological and data framework that can transform quality evaluation, biomanufacturing‐process monitoring, and intellectual‐property protection of probiotics and other live‐cell products. Therefore, we believe this work will attract broad interest and citations.

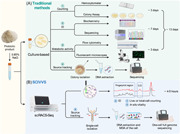

**Abbreviations**
16S rRNA: 16S ribosomal RNA
ACC: accuracy
AFLP: amplified fragment length polymorphism
CDRs: C–D ratios
CNNs: convolutional neural networks
CNVFs: cells number in the five visual fields
DCN: Dilated Convolution Network
EMA: ethidium monoazide
FCM: flow cytometry
FCNs: Fully Convolutional Networks
FNR: false negative rate
FPR: false positive rate
FT‐IR: Fourier Transform Infrared
HI: heterogeneity index
ID: identification
IoU: Intersection‐over‐Union
IP: intellectual‐property
MAL: Metabolic Activity Level
MALDI‐TOF MS: Matrix‐Assisted Laser Desorption/Ionization‐Time of Flight Mass Spectrometry
MAL‐HI: Metabolic Activity Level Heterogeneity Index
MDA: multiple displacement amplification
MLST: multilocus sequence typing
MS/MS: Tandem Mass Spectrometry
OER: overall error rate
PCR: polymerase chain reaction
PMA: propidium monoazide
RAGE: Raman‐activated gravity‐driven single‐cell encapsulation
RFs: Random Forests
rMAL: relative Metabolic Activity Level
SAGs: single‐cell assembled genomes
SCIVVS: Single‐Cell Identification, Viability and Vitality tests, and Source‐Tracking
scRACS‐Seq: single‐cell Raman‐Activated Cell Sorting and Sequencing
SCRS: single‐cell Raman spectrum
SD: standard deviation
SNPs: single‐nucleotide polymorphisms
SNR: signal–noise ratio
SVM: Support Vector Machine

## INTRODUCTION

Probiotics are “living microorganisms that, when administered at appropriate levels, will bring health benefits to the host” [1]. Consumption of probiotics has become a global trend, with many health‐related probiotic products already on market or under development [2, 3, 4, 5, 6, 7]. For manufacturing, consumption, and regulation of such live‐cell products, quality assessment is pivotal. A probiotic product must fulfill standards in quality, safety, and functionality, as those not meeting the standards are associated with loss of beneficial functions or even endangerment of health [8, 9, 10, 11]. Moreover, missing, incompleteness, or inaccuracy of core quality parameters in product declaration would hinder not just informed product selection by consumers but proper regulation by government agencies and statutes [12, 13]. For a given probiotic product, such core quality parameters typically include: (i) total live‐bacteria count, (ii) identification (ID) of ingredient organism(s), (iii) organism‐resolved viability and vitality, and (iv) source‐tracking of the organism(s). However, due to the limitations of existing approaches, a rapid, sensitive, accurate, comprehensive, and generally applicable framework for assessing these quality parameters remains a major technological challenge, and its solution is considered one top priority of the rapidly expanding probiotics industry [14, 15].

Specifically, challenges are profound in each core quality parameter. (i) “Total live‐cell count,” which measures the overall viability of cells [16], can be derived via culture‐based approaches, such as plate counting and agar diffusion; however they can be insensitive, laborious, and time‐consuming [17]. Moreover, plate counting, based on the number of colonies instead of cells [18], is usually unable to account for cells that are sublethally damaged, injured, inhibited, dormant, or inactive [19]. On the other hand, culture‐independent methods, such as polymerase chain reaction combined with ethidium monoazide or propidium monoazide, can also count live/dead cells [16] (based on the prevention of DNA amplification in dead cells), yet the high reagent cost, complexity of operation and inaccuracy due to variation in DNA‐extraction efficiency have limited their industrial application [20].

(ii) “ID of ingredient organism(s)” is a primary prerequisite for documenting the microbiological safety of probiotics products [21, 22]. Genotype‐based ID techniques include those based on 16S ribosomal RNA (16S rRNA) gene sequence, housekeeping genes (e.g., phenylalanyl‐tRNA synthase alpha subunit, RNA polymerase alpha subunit, β‐tubulin, and calmodulin), genome‐wide single‐nucleotide polymorphisms (SNPs), multilocus sequence typing (either core genome or whole‐genome), or amplified fragment length polymorphism [23, 24, 25, 26, 27]. Matrix‐Assisted Laser Desorption/Ionization‐Time of Flight Mass Spectrometry, Fourier Transform Infrared (FT‐IR) spectroscopy, and Tandem Mass Spectrometry (MS/MS) are also frequently used for ID [28, 29, 30, 31, 32, 33]. However, all these methods usually require a culture‐based strain isolation step that can take up to 7 days [22, 25] (and even longer for slow‐growing cells [28]) and thus greatly delay the time to report. Metagenomic approaches are culture‐independent [7], yet due to the need for DNA extraction, library construction, and then sequencing of total DNA, the costs of time and consumables are prohibitive for product quality assessment. Moreover, metagenomics are generally unable to distinguish between free DNA and DNA inside live cells, nor can they assess in situ viability or vitality of cells [7]. Therefore, a fast, culture‐independent, and low‐cost ID approach that either resolves or can couple to the assessment of viability and vitality is highly desirable.

(iii) “Vitality,” which measures the robustness of a live‐cell's metabolic activity in situ, is a concept distinct from “viability” which usually refers to whether the cell is alive or dead [34]. Vitality is also a pivotal quality parameter, since it is directly correlated with probiotic functioning and their health benefits [35]. Employing fluorescent probes, flow cytometry (FCM), and epifluorescence microscopes equipped with high‐quality image acquisition and analysis system can assess the vitality of probiotic products [36, 37]. However, the disadvantages include expensive consumables, complicated operations (especially for inexperienced operators), and analyses that are invasive and inaccurate [35, 36, 37]. Especially, live cells may be hampered or damaged by exposure to light of a fluorescence microscope or fluorescent staining agents, thereby compromising cellular vitality [38]. Additionally, cell wall and cell membrane may affect the entry of fluorescent probes into cells, which reduces the success rate of fluorescent staining and causes inaccuracy in results [39]. Due to these methodological hurdles, vitality (and its heterogeneity) is not yet widely adopted for quality assessment of probiotics, despite its significance to product efficacy. Therefore, rapid, label‐free, accurate methods for evaluating in situ vitality and its heterogeneity in a species‐resolved manner are in urgent demand.

(iv) “Source‐tracking” is a crucial need in the probiotics industry, as proper intellectual‐property (IP) protection of commercial strains is a cornerstone of innovations [40, 41]. Many probiotic strains have been patented or protected, and companies claiming such rights can exclude others from using them in commercial products [42]. Moreover, as genetic exchanges can introduce unintended or even harmful elements such as phages or antimicrobial resistance genes into the original strains, genomic changes of probiotic strains should be monitored [43]. Therefore, source‐tracking is important not just for IP protection but for ensuring product safety. For accurate and reliable tracking of strains found in a product, a full genome sequence is the gold standard, however it can take weeks to obtain, as isolation of the target strain to derive a pure culture of sufficient biomass is usually required before initiating whole‐genome sequencing.

Finally, in addition to the methodological hurdles, one key challenge is to establish an integrated and automation‐prone platform that completes all the quality‐assessment tasks in a streamlined and efficient manner. At present, distinct instruments are usually employed for each of the tasks, for example, culture plates and incubators for live‐cell count, MS for ID, FCM for vitality, and DNA sequencers for source‐tracking, thus integration and automation of workflows become quite difficult. In contrast, an integrated platform that can tackle multiple tasks simultaneously is highly advantageous, as it would facilitate workflow automation and reduce operation costs.

We have introduced the concept of ramanome/meta‐ramanome, the collection of single‐cell Raman spectrum (SCRS) sampled from a cell population or consortium [44], as a kind of single‐cell‐resolution metabolic phenome, and demonstrated the ability of SCRS to rapidly classify microbial species and quantatitively measure metabolic vitality (via tracking D_2_O intake; dead cells exhibit no metabolic activity and therefore do not consume D_2_O) in a culture‐free and label‐free manner [3, 45, 46, 47, 48, 49]. Moreover, by developing a series of Raman‐activated Cell Sorting and Sequencing (RACS‐Seq) techniques, we proved the feasibility of simultaneously obtaining the metabolic phenome and its corresponding high‐coverage genomes at precisely one‐bacterial‐cell resolution and from various types of microbiome samples [44, 50, 51].

Therefore, we proposed a label‐free, single‐cell, genome‐phenome‐combined strategy that exploits the complementary strength of molecular microspectroscopy and genome sequencing, called Single‐Cell Identification, Viability and Vitality tests, and Source‐tracking (SCIVVS), and validated it using lactic acid bacteria including *Lactobacillus*, *Bifidobacterium*, and *Streptococcus* as examples. In SCIVVS, D_2_O‐probed single‐cell Raman microspectroscopy for individual cells directly extracted from the product is employed for total live‐cell counting, organismal identification, and species‐resolved in situ Viability and Vitality test, while single‐cell RACS‐Seq (scRACS‐Seq) is used for full‐genome‐based source‐tracking from just one sorted target cell. By evaluating SCIVVS with actual probiotic products, we demonstrated >20‐fold acceleration and 10‐time cheaper cost, species‐resolved viability and vitality, heterogeneity‐revealing measurements, and high resolution in source‐tracking. Therefore, SCIVVS is a new technological as well as data framework that can transform current practice in quality control, process monitoring, and IP protection of probiotics and other live‐cell products.

## RESULTS

### Overview of the SCIVVS strategy for rapid and comprehensive quality assessment of probiotics

**Figure 1:**
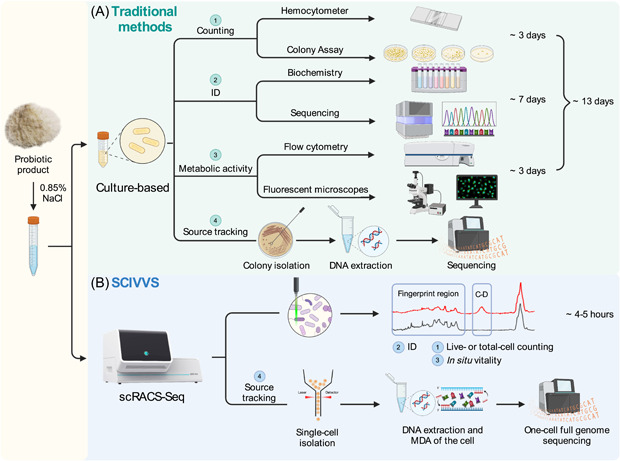
The SCIVVS workflow for total live‐bacteria count, rapid identification, species‐resolved in situ vitality test, and source‐tracking at precisely one‐cell resolution directly from commercial probiotic products. (A) The pure‐culture‐based conventional approach. (B) The culture‐free, single‐cell‐resolution SCIVVS approach. ID, identification; MDA, multiple displacement amplification; SCIVVS, Single‐Cell Identification, Viability and Vitality tests, and Source‐Tracking; scRACS‐Seq, single‐cell Raman‐Activated Cell Sorting and Sequencing.

For a given probiotic product, a complete, traditional quality‐assessment workflow typically consists of four components that are all dependent on strain isolation (Figure 1A): (i) live‐cell counting based on colonies on an de Man, Rogosa and Sharpe (MRS) medium agar plate, which can take at least 3 days; (ii) culture‐based isolation followed by a bacteriological examination‐based ID phase that can take an additional 4 days; (iii) fluorescence‐based vitality assay based on a pure culture that is derived from a colony on plate;(iv) genome sequencing of a pure culture for source‐tracking, which can only start after obtaining a colony on plate. Therefore, the process, even without considering the time consumed for sequencing (which greatly varies, depending on the sequencing platform chosen), usually takes at least a week.

In contrast, SCIVVS consists of three sequential steps which start with cells extracted from the live‐cell product and proceed in a culture‐independent, integrated manner (Figure 1B): (i) treatment of extracted probiotic cells in the liquid MRS medium with 100% D_2_O, then incubation for 3 h for D_2_O intake; (ii) automated SCRS acquisition for the D_2_O‐fed cells, which reports total live‐cell count, ID, and species‐revolved in situ viability and vitality; (iii) sorting of individual cells into microdroplet for precisely one‐cell reaction of multiple displacement amplification (MDA) and whole‐genome sequencing by scRACS‐Seq. As no culture‐based strain isolation is required, the total live‐bacteria count, ID and organism‐resolved in situ viability and vitality can be reported within 5 h (while the time for source‐tracking depends on the sequencing platform).

### Measuring the total live‐cell count directly from probiotic products in SCIVVS

**Figure 2:**
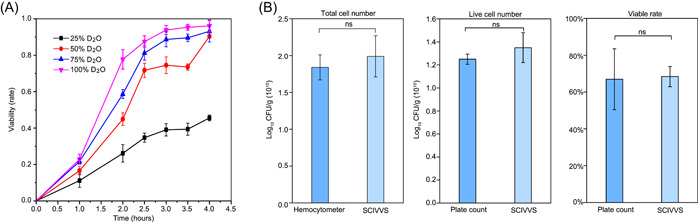
SCRS‐based total live‐bacteria counting of MPP‐A at single‐cell resolution. (A) Intensity of deuterium incorporation in different concentrations of D_2_O MRS media of MPP‐A at different times. The percentages of live cells were labeled with deuterium for 0, 1, 2, 2.5, 3, 3.5, and 4 h. (B) The total‐cell count, live‐cell count, and viable rate of MPP‐A based on the culture‐based plating counting method or the culture‐free SCIVVS. SCIVVS, Single‐Cell Identification, Viability and Vitality tests, and Source‐Tracking; SCRS, single‐cell Raman spectrum.

As a live cell would assimilate D_2_O which results in the emergence a C–D band whose intensity correlates with cellular vitality (via C–D ratio or CDR [49]), we propose a D_2_O‐probed Raman microspectroscopy‐based method for the live‐cell count of probiotic products, where Metabolic Activity Level (MAL) of a cell ($\mathrm{MAL}={\mathrm{CDR}}_{\mathrm{sample}}-{\mathrm{CDR}}_{0h}$; Methods section; Figure S1) indicates whether a cell is viable. To test this hypothesis, we started by selecting a proper D_2_O level under which cell viability can be reliably deduced based on active D_2_O intake as quantified by MAL. The monostrain probiotic product of mono‐strain probiotic product A (MPP‐A) which contains *Lactobacterium plantarum* was employed as a model (Table S1). For each sample, SCRS from about 100 cells were acquired and then compared among various medium D_2_O concentrations (0%, 25%, 50%, 75%, and 100%). At each of the D_2_O levels, due to the gradual formation of C–D bands on newly synthesized biomolecules, a C–D band (2040−2300 cm^−1^) in SCRS would emerge which proportionally increases with time, consistent with active D_2_O intake by live cells (Figure 2A). Moreover, under 100% D_2_O, based on MAL, the proportion of live cells was 22.89% ± 0.04% at 1 h and 93.88% ± 0.03% at 3 h, consistent with time‐dependent D_2_O intake by viable cells. Notably, after the first 3 h, the proportion of live cells did not further increase (Figure 2A).

To evaluate the feasibility of incubation with 100% D_2_O on counting viable cells, we used traditional, culture‐based method to compare the cell counts before and after the 3‐h‐incubation with 100% D_2_O. The live‐cell counts of *L. plantarum*, at 1.26 ± 0.03 × 10^10^ and 1.31 ± 0.05 × 10^10^ colony‐forming unit (CFU)/g (H_0_: *p* > 0.05), respectively, are identical, suggesting that 3‐h‐incubation with 100% D_2_O would not affect live‐cell counts (Figure S2). As for total‐cell count, at 1.84 ± 0.17 × 10^10^ and 2.18 ± 0.21 × 10^10^ CFU/g (H_0_: *p* > 0.05), they are also equivalent, thus the incubation procedure does not affect total‐cell count either (Figure S3). Notably, while 100% D_2_O incubation fully inhibits it, the proliferation of cells that takes place under lower D_2_O concentrations (≤75%) can skew the total‐cell and live‐cell counting results (Figure 2A): for example, the live‐cell counts are 1.27 ± 0.10 × 10^10^ and 2.81 ± 0.09 × 10^10^ CFU/g (H_1_: *p* < 0.05) before and post 3 h 75% D_2_O incubation, respectively, suggesting that choice of 75% D_2_O can overestimate the live‐cell count (Figure S2). Thus, the MAL after 3 h incubation with 100% D_2_O was chosen as the default setting for SCRS‐based live‐cell counting (MAL ≤ 0, dead cells; MAL > 0, live cells).

We then tested the method accuracy using MPP‐A. As the reference, results from the traditional methods were derived in triplicates (Figures 1A and S2): (i) hemocytometer counting for the total *L. plantarum* cell number (averagely 1.84 ± 0.17 × 10^10^ CFU/g; ~1‐h procedure), and (ii) MRS medium agar plate‐based colony counting for the live‐cell number (averagely 1.25 ± 0.45 × 10^10^ CFU/g; ~3‐day procedure) (Figures 1A and S3).

In SCIVVS which takes 4–5 h for the counts, MPP‐A was incubated in the MRS medium with 100% D_2_O anaerobically for 3 h, and then SCRS were acquired to obtain the MAL values (MAL ≤ 0, dead cells; MAL > 0, live cells); then total‐cell count was derived by all the SCRS (MAL ≤ 0 and MAL > 0), while total live‐cell count derived by those SCRS whose MAL values were over 0 (Methods section). The total‐cell and live‐cell counts were calculated as 1.99 ± 0.28 × 10^10^ and 1.35 ± 0.13 × 10^10^ CFU/g, respectively, representing a viable rate of 67.84% (Figure 2B). Clearly, based on SCIVVS, both the total‐cell count and total live‐cell count are equivalent to the hemocytometer counting (H_0_: *p* > 0.05) and the plate‐based colony counts (H_0_: *p* > 0.05; Figure 2B). Therefore, SCIVVS can perform accurate live‐cell counting, directly from probiotic products, in a culture‐free manner.

### Measuring the vitality of various bacterial species at one‐cell resolution directly from probiotic products in SCIVVS

Considering the quantitative nature of CDR in measuring assimilative activity, we hypothesize that besides the viability‐based live‐cell count, “in situ vitality” of individual cells can also be derived from the SCRS that are directly sampled from probiotic products. To test this hypothesis, the relative Metabolic Activity Level (rMAL) was designated and defined as $\mathrm{rMAL}=\frac{{\mathrm{CDR}}_{\mathrm{sample}}-{\mathrm{CDR}}_{0h}}{{\mathrm{CDR}}_{\mathrm{control}}-{\mathrm{CDR}}_{0h}}$ (Methods section), which is the MAL under a particular condition versus the MAL under the optimal condition (i.e., in the log phase for a pure culture of the organism), and represents the relative vitality as compared with peak vitality. For example, for the MPP‐A product, the average MAL and rMAL were 0.0266 ± 0.0019 and 0.9331 ± 0.0016, respectively. Thus, within just 5 h, SCIVVS can quantify the metabolic vitality at single‐cell resolution by MAL, and the relative vitality by rMAL (Figure 2B).

**Figure 3:**
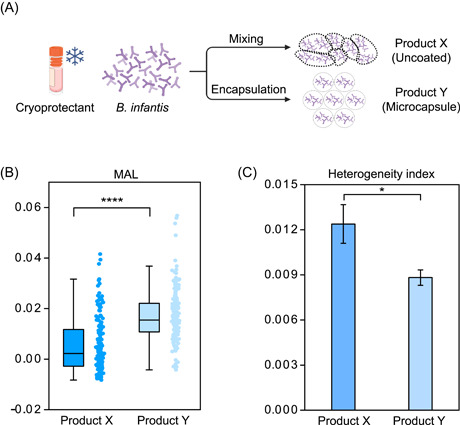
SCRS‐based in situ vitality test of probiotics at single‐cell resolution. (A) The workflow of different processing technologies (either with or without microencapsulation). Also shown are the MAL value (B) and heterogeneity index (HI; C) of Product *X* and Product *Y*, both of which were incubated with 100% D_2_O MRS media for 3 h. MAL, Metabolic Activity Level; SCRS, single‐cell Raman spectrum.

The ability to rapidly measure the vitality at single‐cell resolution can be exploited in many applications. For example, to evaluate the effect of microencapsulation on product vitality, two probiotic products of *X* and *Y* both of the same *Bifidobacterium infantis* strain were manufactured, either without or with microencapsulation (Figure 3A and Table S1). However, measurements based on conventional, culture‐based live‐cell counts, at 3.40 ± 0.36 × 10^9^ CFU/g for Product *X* and 2.51 ± 0.06 × 10^9^ CFU/g for Product *Y*, suggested viability that is essentially equivalent (Table S2). Consequentially, to probe the effect of microencapsulation on product vitality, a time‐consuming acceleration experiment (also known as the stability test [52], in which the two products both undergo incubation at a higher temperature of 37°C for 10 days for simulating cellular stress‐response and thus deriving for vitality) had to be performed, and then live‐cell counts determined again based on the traditional plate colony counting. This process revealed live‐cell counts of 3.57 ± 0.25 × 10^7^ CFU/g for Product *X* and 2.08 ± 0.14 × 10^9^ CFU/g for Product *Y* after the acceleration experiment (Table S2), suggesting survival rates of 1.05% and 82.76%, respectively. Thus, Product *Y* is more stable in viability than Product *X*, which suggests that microencapsulation is effective in preserving product efficacy. Clearly, such a quality‐assessment process, which requires the 10‐day long acceleration experiment plus over 3 days for each culture‐based live‐cell counting both before and after the experiment, is very time‐consuming and inefficient.

In contrast, by profiling vitality in a rapid and quantitative manner, the SCRS‐based method greatly simplifies the assessment process, in that a vitality measurement that takes just 5 h is able to readily distinguish the two products (thus the 10‐day acceleration experiment is no longer unnecessary). Specifically, before any acceleration experiment, the average MAL values of Product *X* and Product *Y* as derived from D_2_O‐probed SCRS were 0.0056 (MAL_ProductX_ = 0.0140 − 0.0084) and 0.0172 (MAL_ProductY_ = 0.0247 − 0.0075), respectively (Figure 3B), revealing over two‐fold higher cellular vitality for *Y* than *X*. In contrast, live‐cell counts of *X* and *Y* (before any acceleration experiments) as measured via SCRS were 3.43 ± 0.18 × 10^9^ and 2.65 ± 0.18 × 10^9^ CFU/g, respectively (Table S2), consistent with the culture‐plate‐based counting results (H_0_: *p* > 0.05). Therefore, by exploiting SCIVVS's ability to measure vitality in addition to viability, the effect of microencapsulation can be quantitatively and more sensitively and efficiently evaluated, i.e., without the need for the time‐consuming acceleration experiment.

Moreover, the degree of intercellular heterogeneity in vitality can be quantitated by the SCRS. We proposed a parameter called heterogeneity index (HI) of MAL (MAL‐HI), defined as the standard deviation (SD) in MAL of individual cells sampled from a probiotic product. Notably, the MAL‐HI_ProductX_ (at 0.012) was significantly higher than the MAL‐HI_ProductY_ (at 0.009), which revealed that the metabolic vitality of individual *B. infantis* cells manufactured with microencapsulation is not just higher on average but more homogeneous (Figure 3C). Therefore, for probiotic products with a largely equivalent live‐cell count, SCIVVS can rapidly reveal distinction in not just the level of metabolic vitality but also their heterogeneity (i.e., the degree of synchronization). This ability to quantify the vitality of probiotics can save the need for performing the very time‐consuming acceleration experiment, as cells of higher vitality can potentially better withstand the challenge of environmental stresses.

### Rapid ID at single‐cell resolution for various bacterial species directly from probiotic products in SCIVVS

**Figure 4:**
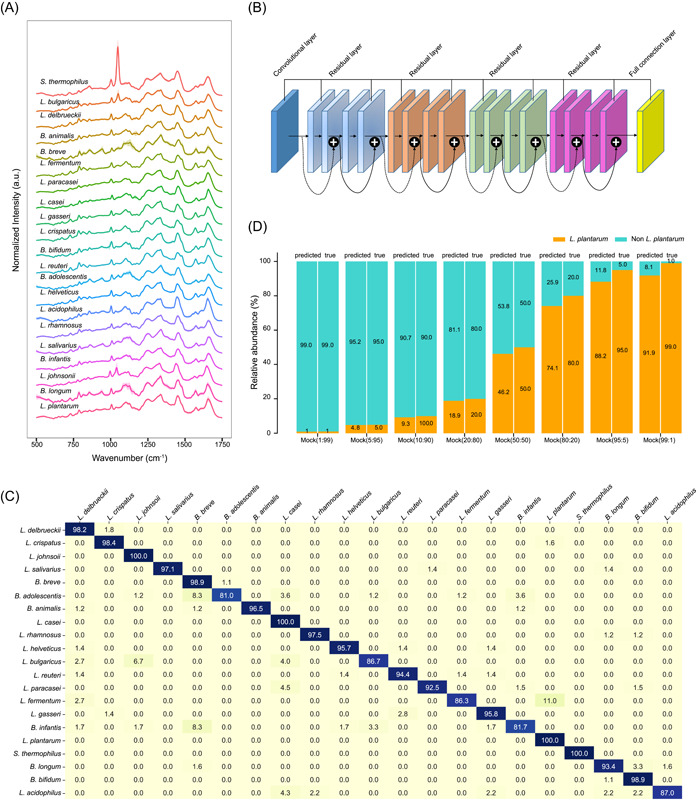
SCRS‐based ID of probiotic cells directly from commercial probiotic products. (A) Average SCRS from the 21 standard statutory strains are shown in bold and overlaid on representative examples of SCRS for each strain. (B) Confusion matrix for 21 strains classes. Entry *i*, *j* represents the percentage out of test spectra that are predicted by the CNN as class *j* given a ground truth of class *i*; entries along the diagonal represent the accuracies for each class. (C) Using a one‐dimensional residual network with 18 total layers, SCRS is classified as one of 21 strains classes. (D) The performance comparison of predicted and true relative abundance of the mock samples which contain different ratios of *Lactobacillus plantarum* 299 V spectra and non‐*L. plantarum* 299 V spectra. *B.*, *Bifidobacterium*; CNN, convolutional neural network; ID, identification; *L.*, *Lactobacillus*; *S.*, *Streptococcus*; SCRS, single‐cell Raman spectrum.

Rapid identification of organism(s) is highly desirable in quality assessment of both mono‐ and multistrain probiotic products, however existing culture‐, MS‐, or metagenomics‐based approaches can take days and be costly. To establish a rapid (i.e., taking minutes) and low‐cost ID method that is not dependent on either culture, MS or sequencing, we started by building a reference database of SCRS from 21 pure‐cultured probiotic strains that represent the standard statutory strains for human consumption (including 14 *Lactobacillus* spp., six *Bifidobacterium* spp., and one *Streptococcus* sp.; Table S3). From each of the pure‐cultured strains, SCRS from over 100 cells was acquired (Figure 4A). Using deep learning models with one‐dimensional spectra as input, our convolutional neural networks (CNNs) architecture consists of 18 layers (17 one‐dimensional convolutional layers and residual connections plus a fully connected layer; Figure 4B; Methods section). Large‐scale convolutions were employed, instead of global average pooling layers, to preserve the exact locations of spectral peaks.

The task of this neural network was configured as a 21‐class classification, where the CNN outputs a probability distribution across the 21 trained‐classes and the maximum was then taken as the predicted class. A training data set of 3546 SCRS and a test data set of 1519 SCRS from the 21 probiotic strains were used as inputs (Table S3). A performance breakdown for individual classes suggests that, on the 21‐class task, the average accuracy of SCRS‐based ID is 93.02% ± 1.39% (±calculated as SD across three validation splits; Figure 4C). This is much higher than Random Forests and Support Vector Machine (at 59.16% and 70.27%, respectively). The CNN model reports an identification accuracy of 93.02% ± 1.39%, thus SCRS is able to reliably distinguish among pure cultures of the 14 *Lactobacillus* spp., six *Bifidobacterium* spp., and one *Streptococcus* sp.

Next, to test the accuracy of this CNN model on species ID, we started with a mock microbiota, in which the *L. plantarum* 299 V and a mixture stock containing equal amounts of the 20 standard statutory probiotic strains were combined in various ratios (1:99, 5:95, 10:90, 20:80, 50:50, 80:20, 95:5, and 99:1; named as Mock‐1, Mock‐5, Mock‐10, Mock‐20, Mock‐50, Mock‐80, Mock‐95, and Mock‐99; Figure 4D). For each of the eight mock probiotic samples, 10 randomized SCRS‐based ID experiments were performed using the CNN model. In each experiment, the proportion of *L. plantarum* 299 V in the mock community was accurately reconstructed (with discrepancy between predicted and actual results <3.5%; Figure 4D). Thus, the model can reliably distinguish *L. plantarum* 299 V single cells from other probiotic cells.

Furthermore, we tested whether this CNN model can identify individual probiotic cells directly from the MPP‐A product. Notably, for this mission, the reference database needs to be optimized by adding SCRS of *L. plantarum* 299 V from MPP‐A (Table S1). Specifically, MPP‐A was incubated in an MRS medium with 100% D_2_O for 3 h. Then the supernatant of the incubated sample was divided into three aliquots, respectively. As controls, two aliquots underwent 16S‐rDNA‐based amplicon sequencing and metagenome sequencing, respectively, which both pinpointed *L. plantarum* as the major ingredient. The other aliquot was washed twice and individual cells were analyzed for SCRS‐based ID, where on average 92.72% of SCRS were predicted as *L. plantarum* by the CNN model. As the three methods all produced results that support *L. plantarum* as the only dominant species in MPP‐A (Figure 4 and Table S4), we conclude that the SCRS‐based CNN model can accurately identify *L. plantarum* single cells directly from actual probiotic products, in the absence of pure culture.

### Source‐tracking of single cells in probiotic products via one‐cell genome sequences using scRACS‐Seq in SCIVVS

**Figure 5:**
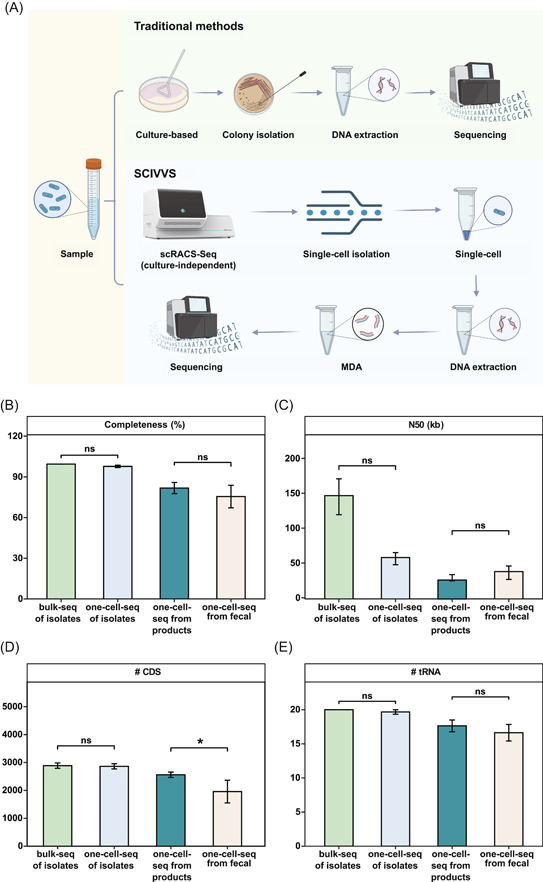
Source‐tracking at precisely one‐bacterial‐cell resolution directly from commercial probiotic products by scRACS‐Seq. (A) The scRACS‐Seq workflow for the source‐tracking of commercial probiotic products compared with the traditional, cultured‐based method. SCIVVS for a pure culture of *Lactobacillus* spp. and genomic sequencing of bulk samples were also compared. For the pure culture of *Lactobacillus* spp. (“pure”), three cells (B1, B3, and B4) were individually sorted and sequenced from precisely one cell. For probiotic products, 16 cells (C1, C2, C3, C4, C5, C6, C7, C8, C9, C10, C11, C12, C13, C14, C15, and C16) were individually sorted and sequenced from precisely one cell. (B) Completeness of the SAGs based on CheckM estimation. (C) Continuity of de novo assemblies based on N50 length. (D) Number of CDS regions mined from de novo assemblies. (E) Number of tRNA types mined from de novo assemblies. CDS, protein‐coding sequence; MDA, multiple displacement amplification; SAG, single‐cell assembled genome; SCIVVS, Single‐Cell Identification, Viability and Vitality tests, and Source‐Tracking; scRACS‐Seq, single‐cell Raman‐Activated Cell Sorting and Sequencing; tRNA, transfer RNA.

As the acquisition of SCRS is nondestructive, we propose that individual bacterial cells directly extracted from probiotic products can directly undergo source‐tracking, based on RACS and then one‐cell whole‐genome sequencing (scRACS‐Seq; Figure 5A). To establish this method, we started by employing *Lactobacillus rhamnosus* which is one of the strains in compound probiotic product A (CPP‐A) as a model (Table S1).

For benchmarking, Sample A consisting of *L. rhamnosus* cells was derived. Via a Raman‐activated gravity‐driven single‐cell encapsulation (RAGE) chip that we introduced [51], the cells were individually sorted in a microdroplet, lysed and amplified for DNA via MDA at one‐cell resolution (Methods section). Then the cells of A1–A4 (all from Sample A) and O5 (the empty droplet without any cells; as negative control) were sequenced for the 16S rDNA gene, which revealed that the cells A1–A4 were all *L. rhamnosus* (Figure S4A and Table S5). Moreover, the A1 cell was shotgun sequenced, and the 3.03 million read pairs were assembled into a near‐complete genome (completeness of 93.53% and contamination of 1.84%; Table 1). Genome alignment pinpointed the A1 cell as *L. rhamnosus*, consistent with the 16S rRNA‐based results.

**Table 1:** Precise one‐cell genome sequencing of single bacterial cells directly from commercial probiotic products via the scRACS‐Seq approach.

Cell ID	Read coverage (*X*)	Completeness (%)	Contamination (%)	GC (%)	Total length (Mbp)	Strain
The A1 cell	269.56	93.53	1.84	46.87	3.03	*L. rhamnosus*
The B1 cell	220.30	97.53	1.91	46.50	2.99	*L. rhamnosus*
The B3 cell	359.86	96.58	4.41	44.66	3.41	*L. plantarum*
The B4 cell	271.70	99.40	1.25	46.03	3.21	*L. paracasei*
The C1 cell	325.56	97.62	0.57	46.27	3.06	*L. paracasei*
The C2 cell	321.51	99.07	0.37	46.23	3.06	*L. paracasei*
The C3 cell	416.78	89.95	3.31	44.54	2.87	*L. plantarum*
The C4 cell	282.76	89.14	5.43	46.23	3.06	*L. paracasei*
The C5 cell	316.42	48.2	5.17	45.97	1.92	*L. paracasei*
The C6 cell	379.77	80.25	5.35	44.43	2.85	*L. plantarum*
The C7 cell	336.19	70.77	4.19	44.49	2.65	*L. plantarum*
The C8 cell	381.30	95.12	0.91	46.23	2.94	*L. paracasei*
The C9 cell	303.57	68.68	6.11	44.26	2.39	*L. plantarum*
The C10 cell	320.17	46.28	6.71	46.14	1.77	*L. paracasei*
The C11 cell	291.14	90.72	3.44	44.49	3.04	*L. plantarum*
The C12 cell	381.05	78.04	2.29	46.19	2.62	*L. paracasei*
The C13 cell	314.16	82.62	8.05	44.52	3.09	*L. plantarum*
The C14 cell	438.33	97.01	0.72	46.27	3.01	*L. paracasei*
The C15 cell	444.06	79.33	8.20	44.50	3.00	*L. plantarum*
The C16 cell	370.59	96.74	3.73	46.27	3.07	*L. paracasei*

*Note*: For Sample B, we have sorted 15 single cells in a one‐cell‐one‐tube manner, among which three cells each representing a distinct bacterial species were selected for whole‐genome sequencing and were shown here.

Abbreviations: GC%, guanine‐cytosine content; ID, identification; *L. paracasei*, *Lactobacillus paracasei*; *L. plantarum*, *Lactobacillus plantarum*; *L. rhamnosus*, *Lactobacillus rhamnosus*.

Next, we designed a mock microbiota (*L. paracasei* vs. *L. rhamnosus* vs. *L. plantarum*; 1:1:1, all from CPP‐A) to obtain Sample B. Then the cells B1–15 (from Sample B) and O16 (the empty droplet without any cells; as negative control) were individually sorted in a microdroplet, lysed and amplified for DNA and sequenced. The one‐cell 16S rRNA gene sequences pinpointed B3, B6, and B10 as *L. plantarum*, B4, B7, and B9 as *Lactobacillus paracasei*, and B1, B8, B11, and B13 as *L. rhamnosus* (Figure S4B and Table S5). For the B1, B3, and B4 cells, shotgun sequencing produced 2.99, 3.41, and 3.21 million read pairs (via the HiSeq platform), which were each assembled into near‐complete single‐cell assembled genomes (SAGs), with 97.53%, 96.58%, and 99.40% in completeness and 1.91%, 4.41%, and 1.25% in contamination, respectively (Table 1). These high‐quality one‐cell genomes pinpointed the B1, B3 and B4 cells as *L. rhamnosus*, *L. plantarum*, and *L. paracasei*, consistent with the one‐cell 16S rRNA‐based ID results (Table 1).

To test how the quality of such precisely one‐cell‐derived SAGs compared with the conventional, pure‐culture‐derived genomes, the bulk cultures of the *L. paracasei*, *L. plantarum*, and *L. rhamnosus* strains isolated via plating of CPP‐A were also shotgun sequenced and assembled, which show an average N50 of 148.28 Kbp and estimated genome completeness at 99.43% ± 0.05% (Figure 5B–E). Notably, all the three SAGs are of quality comparable to the pure‐culture‐derived assemblies (Table 1 and Figure 5B–E). Therefore, SCIVVS can produce high‐quality one‐cell genome sequences for reliably source‐tracking strains directly from probiotic products at single‐cell resolution.

### An automated, integrated SCIVVS workflow for quality assessment of multistrain probiotic products

**Figure 6:**
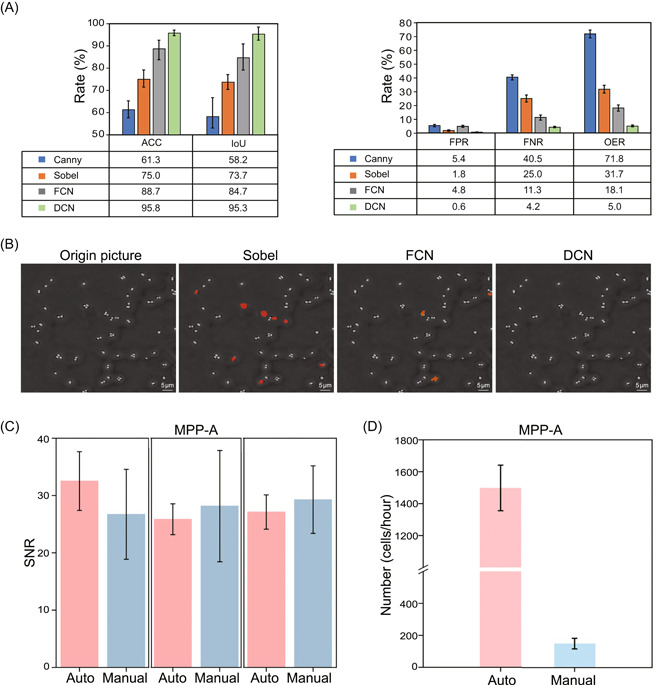
Automated SCRS acquisition for probiotics quality assessment via SCIVVS. (A) Segmentation comparison under different algorithms. Higher ACC and IoU represent more accurate segmentation. By contrast, the smaller of FPR, FNR, and OER means the better segmentation results. (B) Diagram of typical segmentation results for *Lactobacillus plantarum* cells by applying algorithms of Sobel, FCN, and DCN. The red marks in the image indicate errors in segmentation. The advantages of the automated acquisition are apparent, based on comparison with the manual operation (via expert users), for two performance parameters including SNR (C) and throughput (D). ACC, accuracy; DCN, Dilated Convolution Network; FCN, Fully Convolutional Network; FNR, false negative rate; FPR, false positive rate; IoU, intersection‐over‐Union; OER, overall error rate; SCIVVS, Single‐Cell Identification, Viability and Vitality tests, and Source‐Tracking; SCRS, single‐cell Raman spectrum; SNR, signal–noise ratio.

In SCIVVS, live‐cell count, ID, and species‐resolved viability and vitality are all derived from SCRS, thus automation of SCRS acquisition is a top priority in improving SCIVVS throughput. To achieve this, we tested various computational methods for intelligent single‐cell segmentation and localization, and compared the results (Figure 6A). The accuracy of Canny and Sobel in image segmentation is <80% (Canny, ACC = 61.3%; Sobel, ACC = 75%). These methods use threshold judgment and gradient calculation to perform edge detection, which is greatly affected by image quality; moreover, determining the threshold value is difficult, which needs to be adjusted based on image quality. Thus, they are only suitable for segmenting sparse and clear cell images, but not dense blurred cell images. Fully Convolutional Networks, a supervised learning segmentation method, shows 88.7% accuracy. However, in the pooling process, it loses a large amount of information which reduces segmentation accuracy. Dilated Convolution Network (DCN, Methods section) achieves the highest performance (95.8% accuracy), and is especially effective for images with impurities. In DCN, impurity pixels in the image are labeled as the background and assigned with low weight to aid accurate classification; moreover, cell adhesion is greatly reduced, which further refines segmentation results and improves accuracy (Figure 6B).

The automated SCRS‐acquisition procedure thus includes image focusing, single‐cell segmentation and localization, SCRS collection, and quality assessment plus screening. To evaluate its performance, SCRS from the MPP‐A product (Table S1) was collected by both automated acquisition and the manual method. For signal–noise ratio, no significant difference is found between the methods, suggesting equivalent spectral quality (Figure 6C). However, the automated method accelerates data collection by averagely 10.9‐folds, for example, 9.5‐, 12.6‐, and 10.6‐folds for Data sets A, B, and C (with each data set in triplicate experiments; Figure 6D). Therefore, our automated procedure can replace the tedious manual SCRS collection process.

Next, we validated the automated SCIVVS workflow on commercial probiotic products, using the multistrain CPP‐A (Table S1) as an example.

**Figure 7:**
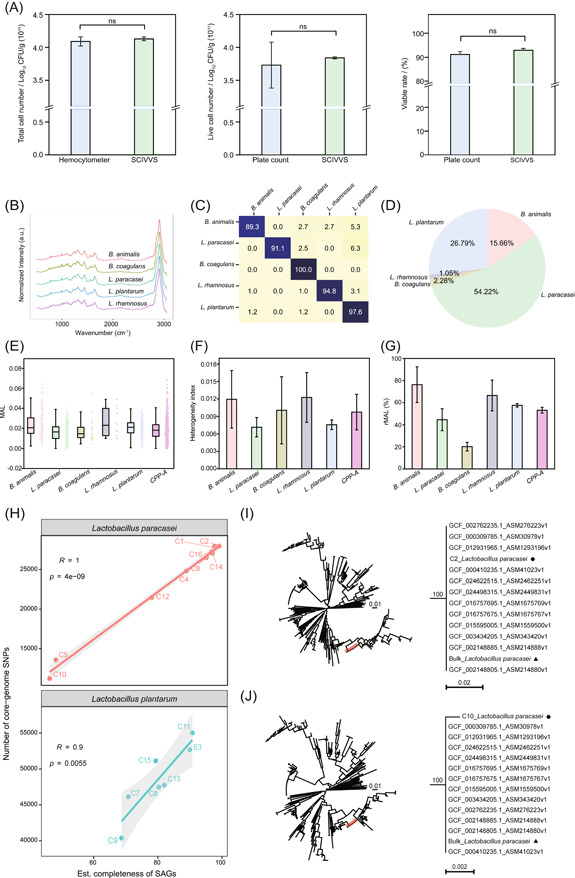
An integrated single‐cell SCIVVS workflow for quality assessment of compound probiotic products. (A) The total‐cell count, live‐cell count, and viable rate of CPP‐A as assessed by plating‐based counting or SCIVVS. (B) Average Raman spectra from five kinds of pure probiotic strains (*Lactobacillus plantarum*, *Lactobacillus paracasei*, *Bacillus coagulans*, *Bifidobacterium animalis*, and *Lactobacillus rhamnosus*) from CPP‐A are shown in bold and overlaid on representative examples of SCRS for each strain. (C) Confusion matrix for five strains classes. (D) The predicted proportion of live strains in CPP‐A. (E) Metabolic Activity Level (MAL) of CPP‐A and each live strain in CPP‐A. (F) The heterogeneity index (HI) of CPP‐A and each live strain in CPP‐A. (G) Relative Metabolic Activity Level (rMAL) of CPP‐A and each live strain in CPP‐A. From real probiotic products, 16 single cells (C1, C2, C3, C4, C5, C6, C7, C8, C9, C10, C11, C12, C13, C14, C15, and C16) were sorted and sequenced via the SCIVVS approach. (H) Relationship between the number of core‐genome SNPs and the completeness of SAGs. The phylogenetic trees reconstructed using those SNPs of the C2 cell (an *L. paracasei* cell with the highest coverage; (I)), or of the C10 cell (an *L. paracasei* cell with the lowest coverage; (J)) are shown. The maximum likelihood trees were constructed using FastTree based on Jukes–Cantor distances of concatenated SNP sites. The bootstrap confidence values (as percentages) are indicated above each branch. The scale bar represents a Jukes–Cantor distance of 0.02 or 0.002. Single cells from real probiotic products are indicated by black circles; bulk‐culture‐based sequencing results are indicated by black triangles. Both high‐coverage and low‐coverage SAGs are clustered with the reference genome in the same phylogenetic branch. SAG, single‐cell assembled genome; SCIVVS, Single‐Cell Identification, Viability and Vitality tests, and Source‐Tracking; SNP, single‐nucleotide polymorphism; SNR, signal–noise ratio.

*Step 1. Total live‐cell counting*: CPP‐A was exposed to 100% D_2_O in an MRS medium for various durations to determine the optimum incubation time. As expected, the proportion of live cells has reached 96.33% ± 0.009% at 3 h and did not increase afterwards (Figure S5). Therefore, for CPP‐A, the MAL after 3 h 100% D_2_O incubation was chosen as default for live‐cell counts. Thus, the total‐cell and total live‐cell counts are 4.13 ± 0.03 × 10^11^ and 3.84 ± 0.02 × 10^11^ CFU/g, respectively. Moreover, 92.98% of the cells are alive while the rest (7.02%) dead. The SCIVVS results are consistent with hemocytometer counting (total‐cell number of 4.09 ± 0.07 × 10^11^ CFU/g; H_0_: *p* > 0.05) and plating‐based live‐cell counts (3.72 ± 0.35 × 10^11^ CFU/g; H_0_: *p* > 0.05; based on those from each of the five pure cultures before mixture into CPP‐A; Figure 7A).

*Step 2. Strain identification*: First, the five ingredient strains in CPP‐A, including *L. plantarum*, *L. paracasei*, *Bacillus coagulans*, *Bifidobacterium animalis*, and *L. rhamnosus*, were used to build a reference SCRS database, in that cells in each pure culture were incubated with 100% D_2_O for 3 h and the SCRS acquired via 532 nm laser (Figure 7B). The ID task was configured as a five‐class classification, where the CNN outputs a probability distribution across the five trained‐classes and the maximum was then taken as the predicted class. A training data set of 1602 SCRS and a test data set of 401 SCRS were used as inputs (Table S6). A performance breakdown for individual classes suggests that, on the five‐class task, the average accuracy of SCRS‐based ID is 94.93% ± 0.01% (±calculated as SD across three validation splits; Figure 7C). Thus, the model offers ID accuracy of 94.93% ± 0.01%, suggesting the ability to reliably distinguish among the five strains in CPP‐A.

To test the feasibility of ID directly from products, CPP‐A was incubated with 100% D_2_O for 3 h and ~400 SCRS acquired directly from the mixture of cells extracted from CPP‐A. Using the CNN model above, the predicted ratios of live cells for the five strains in CPP‐A are 26.79% ± 0.01%, 54.22% ± 0.03%, 2.28% ± 0.01%, 15.66% ± 0.01%, and 1.05% ± 0.01%, respectively (Figure 7D and Table S7). The expected live‐cell proportions of *L. plantarum, L. paracasei, B. coagulans, B. animalis*, and *L. rhamnosus* are 26.24%, 54.10%, 2.02%, 17.08%, and 0.56%, which are based on plating‐based live‐cell counts from each of the five pure cultures before the mixture into CPP‐A. In the three parallel experiments, *χ* ^2^ are 0.9827, 9.9214, and 2.3264, suggesting high consistency between the predicted proportions and the actual ones (if *χ* ^2^ is ≤11.070, the two sets are considered as consistent), despite the wide dynamic range (~100 folds) of relative proportion among the strains. Therefore, our SCRS‐based ID for the five strains in CPP‐A is accurate.

*Step 3. Species‐resolved live‐cell counts, vitality assessment, and heterogeneity of vitality*: On the basis of the SCRS‐based ID for each of the probiotic cells sampled, the distributions of live‐cell count and vitality were derived for each of the five strains in CPP‐A. Specifically, the live‐cell counts are 1.03 ± 0.04 × 10^11^, 2.08 ± 0.10 × 10^11^, 8.76 ± 0.03 × 10^9^, 6.01 ± 0.05 × 10^10^, and 4.03 ± 0.02 × 10^9^ CFU/g, respectively, for the five ingredient strains (Table S7).

Moreover, based on the MAL, HI, and rMAL concepts, the metabolic vitality (both absolute and relative) and the heterogeneity of vitality were revealed, both for product‐overall and for each of the five live strains in CPP‐A (Figure 7E–G): MAL_CPP‐A_ = 0.02, rMAL_CPP‐A_ = 0.53, and HI_CPP‐A_ = 0.01. On the other hand, the live‐cell rates measured by SCIVVS are consistent with those by traditional methods (Table S7). Thus, SCIVVS can accurately quantify these fundamental phenotypic properties at single‐cell resolution and in a strain‐resolved manner, directly from compound probiotic products.

*Step 4. Source*‐*tracking*: For this task, cells extracted from CPP‐A were individually sorted in a microdroplet, lysed and amplified for whole‐genome sequencing via one‐cell RAGE‐Seq, where the whole sorting process takes place in an aquatic phase to protect cell vitality and genome integrity [51]. Specifically, the C1–16 cells from CPP‐A and O11 (the empty droplet without any cell as negative control) were individually sorted, lysed and then amplified in a one‐cell‐per tube manner (Table 1; Methods section). One‐cell 16S rRNA gene sequencing revealed C3, C6, C7, C9, C11, C13, and C15 from CPP‐A as *L. plantarum*, C1, C2, C4, C5, C8, C10, C12, C14, and C16 as *L. paracasei*, both the main ingredients in CPP‐A (Table S5). The corresponding one‐cell whole‐genome shotgun sequencing revealed the high quality of the one‐cell SAGs (Figure 5B–E): (i) the average completeness is 81.79%, that is, no significant difference (Wilcoxon test; *p* > 0.05) with the single probiotic cells isolated with gel beads from mouse fecal (the “SAG‐gel” platform [53]); (ii) contiguity of the SAGs, based on contig N50, from the two platforms are similar (Wilcoxon test; *p* > 0.05); (iii) SAGs from SCIVVS recovered significant more protein‐coding genes than SAGs from SAG‐gel platform (averagely 2561 vs. 1958; Wilcoxon test, *p* < 0.05); (iv) the recovered tRNA gene types from the two platforms are similar (Wilcoxon test, *p* > 0.05). Therefore, SCIVVS not only obtained the metabolic phenotype of each cell, but also produced SAGs with quality comparable to the SAG‐gel platform [53].

The one‐cell SAGs derived by SCIVVS enabled high‐resolution genome comparisons among *Lactobacillus* strains (Table 1). To test the possibility of source‐tracking based on SNP profiles of SAGs, a genome database (“LpaDB”) was constructed which includes the pure‐culture derived genomes of *L. paracasei* from CPP‐A and 283 *L. paracasei* genomes retrieved from the NCBI RefSeq database (Methods section). In LpaDB, ~78% genomes share fully identical 16 S sequences, suggesting 16S‐rDNA‐based phylogeny is of insufficient resolution for source‐tracking in such scenarios. To test whether sufficient SNPs can be obtained for distinguishing the strains, core‐genome SNPs were profiled for the SCIVVS‐derived SAGs individually using Parsnp [54]. The number of core‐genome SNPs is positively correlated with the completeness of SAGs (Figure 7H). For the high‐coverage SAG of C2 (with 99.07% coverage), 27,961 core‐genome SNPs were obtained; the phylogenetic tree reconstructed using these SNPs reveals that the *L. paracasei* SAG of C2 directly sorted from CPP‐A was clustered correctly with the reference which is the pure‐culture‐derived *L. paracasei* genomes (Figure 7I), indicative of their identical origin. Notably, for the lowest‐coverage SAG of C10 (with 46.28% coverage), the *L. paracasei* genome of C10 was also precisely placed at the phylogenetic tree (Figure 7J). For SAGs of *L. plantarum*, at the one‐cell genome coverage of 68.68%–90.72%, accurate source‐tracking results were also obtained (Table 1 and Figure S6). Therefore, the one‐cell SAGs derived by scRACS‐Seq directly from cell extracts of a probiotic product, regardless of the cultivability of strain, are sufficient to support sensitive and reliable source‐tracking.

On the basis of our estimate of experimental duration and consumable costs, SCIVVS takes ~5 h and $4.39 for a comprehensive quality‐assessment process that includes live‐cell count, ID, and species‐resolved in situ viability and vitality (Table S8), and takes an additional ~10 h and $1.12 for DNA preparation for source‐tracking (single‐cell sorting, lysis, and MDA reaction). In contrast, the traditional method would take 9–11 days and $33.31–$58.61 for live‐cell count, ID, and vitality test (via cytometry), and an additional ~5 days and $3.90 for DNA preparation for source‐tracking (plating, colony picking, liquid culture, and then DNA extraction from bulk culture). As a result, SCIVVS can be >20‐time faster and one order of magnitude lower in consumable costs (Table 2).

**Table 2:** Comparison of quality‐assessment parameters for probiotic products between the traditional methods and the SCIVVS method.

Methods	Cost ($)	Duration
*Traditional methods*		
Counting		
Hemocytometer	3.35	3 days
Plate colony	7.16	
ID		
Biochemical	9.35	3–5 days
Sequencing	8.49	
In situ vitality		
Flow cytometry	42.10	3 days
Fluorescent microscopes	21.47	
Source‐tracking		
Culture and then bulk‐cell genome sequencing	3.90^a^	>5 days^a^
*SCIVVS method*		
Counting + ID + in situ vitality		
SCRS‐based methods	4.39	5 h
Source‐tracking		
Single‐cell sorting and then sequencing	1.12^b^	>10 h^b^

Abbreviations: ID, identification; MDA, multiple displacement amplification; SCIVVS, Single‐Cell Identification, Viability and Vitality tests, and Source‐Tracking; SCRS, single‐cell Raman spectrum.

^a^ Cost or duration for the three steps including plating, colony picking and liquid culture, and DNA extraction.

^b^ Cost or duration for the two steps including single‐cell sorting directly from sample, and then reaction for cell lysis and MDA. As the cost or duration for genome sequencing depends on the specific sequencing platform chosen, the estimates for source‐tracking do not include the sequencing step.

## DISCUSSION

A comprehensive approach such as SCIVVS that integrates rapid live‐bacteria count, ID, in situ vitality test, and genome‐based source‐tracking at the ultimate resolution of a cell can tackle the pressing challenges in quality inspection of probiotic products. Instead of waiting for pure cultures and counting colonies on plate, SCIVVS exploits the information‐rich D_2_O‐probed SCRS acquired directly from products: the fingerprint region enables species‐level classification of the cell with high accuracy based on a reference SCRS database of common probiotic species, whereas the C–D band accurately quantifies both total live‐cell count and metabolic vitality by deriving absolute and relative MAL. Moreover, for source‐tracking of target strains, scRACS‐Seq can further proceed, which produces indexed, precisely one‐cell‐based genome assemblies that can reach >99% genome‐wide coverage for the various *Lactobacillus*, *Bifidobacterium*, and *Streptococcus* spp. Furthermore, we demonstrated an integrated SCIVVS workflow with automation in SCRS acquisition, and validated the workflow with actual mono‐ and multistrain probiotic products.

In SCIVVS, the integration of Raman microspectroscopy and single‐cell sorting, lysis, and DNA amplification in a RAGE chip [51] allows the direct coupling of SCRS‐based, noninvasive metabolic phenome profiling to downstream single‐cell genome sequencing. This design thus takes full advantage of, (i) the high speed and throughput of the former in rapid ID plus vitality and viability tests, and (ii) the high resolution of the latter in source‐tracking. Arguably, the metabolic‐phenome‐guided single‐cell sequencing approach like SCIVVS can be the most efficient, in terms of both time and consumable costs, for circumstances such as live‐cell product quality assessment, since speed and throughput are the priority when processing large sample volumes, and resolution in genome‐based applications such as source‐tracking is only applied to selected samples.

Despite its strength, further developments of SCIVVS are required to test and extend its full potential. For example, this study included only 21 standard statutory strains of probiotics (which are all from lactic acid‐producing bacterial species) as the reference ramanome database for ID, and whether this approach can be extended to other probiotics such as bacillus, propionibacterium, and yeast is not clear. Moreover, as SCRS is sensitive to both the taxonomy and physiological state of a cell, to what degree SCRS can reliably predict taxonomy when the number of species or strains in the probiotics product further increases remains to be addressed, although our previous study on over two dozen microalgal species supported the possibility of distinguishing both taxonomy and state of the cell via SCRS [55]. Therefore, the reference SCRS database should be expanded to include more species and more physiological states, so that the scope and the resolution of SCRS‐based species identification can be fully tested. Moreover, by adopting Raman flow cytometry (FCM) such as FlowRACS [56], the speed of SCRS acquisition can be further elevated. At the sorting step, new designs of the RAGE chip that improve the precision of cell capture, such as optical tweezer‐assisted pool‐screening or the use of artificial intelligence for automated sorting of cells [57, 58], can also enhance the throughput of SCIVVS. Furthermore, techniques that allow barcoded single‐cell genome sequencing are necessary for the high‐quality yet cost‐efficient sequencing of a larger number of sorted cells in parallel [50].

The ability to perform quality assessment of live‐cell products at single‐cell resolution is of profound implications. For example, since one cell in the original sample corresponds to one colony (~10^9^ cells) on the agar plate, the one‐cell phenome‐genome profiling in SCIVVS can directly skip the time‐consuming cell isolation and pure culture, yet without sacrificing any resolution (at least theoretically); instead, it can provide a more accurate picture of diversity and structure for both genotypes and phenotypes of the original sample, since the insertion of a plate‐based isolation and culture step can skew or even misrepresent such pictures due to the variation in growth rate among species. Specifically, since the SCIVVS strategy can detect all the viable cells that include not only active but also dormant (i.e., inactive yet still alive) cells, while the traditional agar‐plate counting method would favor just those active or fast‐growing cells, theoretically SCIVVS can count more cells, and produce a more accurate picture of the sample. This can be important, especially for the accurate detection and counting of pathogenic bacteria from food products (or other types of samples) where current quality standards from regulatory agencies have usually based on the agar‐plate‐based CFU [59]. Therefore, such single‐cell technologies that profile metabolic phenome and genome in situ in an integrated manner will usher in new standards for probiotics product quality assessment. Such standards are particularly timely for the industry, considering commercial probiotic products from various regions such as the United States, China, Italy, and Bulgaria have been assessed, yet regulatory standards have not been widely formulated due to differences between countries [15] and the lack of accurate methods ranging from strain identification to phenotype profiling [60, 61]. In fact, a revision of the quality concept towards a more comprehensive approach, as well as the introduction of tools to valorize beneficial high‐quality probiotic preparations, is called for in Europe [15]. Therefore, the single‐cell metabolic phenomics and genomics based on SCIVVS will become an advantageous quality‐assessment approach, as well as a universally applicable type of quality‐assessment database, to support a new generation of standards for quality control, process monitoring, and IP protection of probiotics and other live‐cell products.

## CONCLUSION

The SCIVVS approach is a comprehensive and efficient solution for the quality inspection of probiotic products, offering rapid live‐bacteria count, ID, in situ vitality testing, and genome‐based source‐tracking at the ultimate resolution of one‐bacterial cell. It takes full advantage of the high speed and throughput of Raman microspectroscopy in live‐bacteria count, ID and in situ vitality testing, and the high resolution of targeted single‐cell genome sequencing in source‐tracking, thus it is suitable for processing the large sample volume required in an industrial production setting. As it is >20‐fold faster, >10‐time cheaper, vitality‐revealing, heterogeneity‐resolving, and automation‐prone, SCIVVS is also a new technological and data framework for quality assessment of live‐cell products that include not just probiotics but also human, animal, higher plant, and algal cell preparations.

## METHODS

### Probiotic strains and products

Twenty‐one standard strains of probiotics were tested in this study (Table S3). They are listed in the <Qualified Presumption of Safety for Microorganisms> approved by EFSA in 2007 [62]. These species can also be used for food in China according to the <Inventory of microbial cultures that can be used in food> which was approved by Chinese Ministry of Health in 2010 [63]. All strains were purchased from China Center of Industrial Culture Collection (CI), except the DSM 20555, DSM 20072, and DSM 9843 which were from the Deutsche Sammlung von Mikroorganismen und Zellkulturen (DE) and the BNCC 341709 which was from BeNa Culture Collection (CN). MRS and BS culture broth were both from Hope Bio‐Technology Co. (CN). Saline and Tween 20 were from Sangon Biotech Co. (CN). D_2_O used for the deuterium labeling of cells was from Sigma‐Aldrich Co. (US). The MRS medium dissolved with D_2_O was filtrated via a 0.22‐μm polyethersulfone membrane filter (US).

MPP‐A from Renhe Group (CN) which is a single‐strain (*L. plantarum* 299 V) product (Catalog#: 6973601560317) was purchased from Jingdong (CN). CPP‐A, a mixture of five probiotic strains (*L. plantarum*, *L. paracasei*, *B. coagulans*, *Bifidobacterium longum*, and *B. infantis*), was provided by Qingdao Eastsea Pharma Co., Ltd. (CN).

### The process of probiotic product quality control based on SCIVVS

All SCRSs were acquired on a RACS‐Seq instrument (Qingdao Single‐cell Biotechnology), as described previously [51]. For single‐cell rapid ID, a reference SCRS database was constructed first, and individual cells were classified based on the database using a CNN. Metabolic vitality and live‐cell counting were measured based on the C–D bond from 2040 to 2300 cm^−1^ in SCRS, via MAL and rMAL [49]. Single‐cell source‐tracking was achieved by MDA and sequencing of RACS‐sorted probiotic cells in a one‐cell‐one‐tube manner via the RACS‐Seq instrument [51]. Full details of the methodology are provided in Supporting Information Methods section.

## AUTHOR CONTRIBUTIONS

Jia Zhang and Jian Xu designed the research. Jia Zhang, Teng Xu, Xiaohang Wang, Mingyue Xu, Ying Li, and Cheng Guo performed the experiments. Jia Zhang, Lei Zhang, Lihui Ren, Yanhai Gong, and Shiqi Zhou performed automation, sequencing and data analysis for single‐cell genomes. Bo Ma and Jian Xu introduced and validated the scRACS‐Seq method and chip. Cheng Guo and Gaishuang Shang provided the probiotic products. Lei Zhai, Xuejian Yu, and Cheng Guo provided several of the pure culture strains. Jia Zhang, Teng Xu, and Jian Xu analyzed data and interpreted results. Xuejian Yu, Gongchao Jing, Pengfei Zhu, Rongze Chen, Chen Wang, Xiaoyan Jing, Changkai Niu, Yunlong Cui, Yuanyuan Ge, and Su Yao provided critical suggestions. Jian Xu, Jia Zhang, Lei Zhang, Yanhai Gong, and Lihui Ren wrote the manuscript.

## CONFLICT OF INTEREST STATEMENT

Jian Xu and Bo Ma are on the scientific board of Qingdao Single‐Cell Biotech. Co., Ltd. No other competing interest is declared.

## Supporting information

Supporting information.

## Data Availability

The sequence data reported in this study were deposited to NCBI SRA database (BioProject ID: PRJNA917206; https://www.ncbi.nlm.nih.gov/bioproject/PRJNA917206). Supporting Information (figures, tables, scripts, graphical abstract, slides, videos, Chinese translated version, and updated materials) may be found in the online DOI or iMeta Science http://www.imeta.science/.
