# Supplementary material for: Single‐cell rapid identification, in situ viability and vitality profiling, and genome‐based source‐tracking for probiotics products
Source: Imeta. 2023 May 25;2(3):e117. doi: 10.1002/imt2.117 (PMC10989769; doi:10.1002/imt2.117)
Supplement: Supplementary file 1 — Supporting information. [file IMT2-2-e117-s001.docx]

**Supplementary information to:**

Title: **Single-cell rapid identification, *in situ* viability and vitality profiling, and genome-based source tracking for probiotics products**

Running Title: **Single-Cell Phenome-Genome Analysis for Probiotics Quality Assessment**

Jia Zhang^1,5,6,7^, Lihui Ren^1,5,6,8^, Lei Zhang^1,5,6,9^, Yanhai Gong^1,5,6,7^, Teng Xu^1,5,6,7^, Xiaohang Wang^1,5,6,7^, Cheng Guo^2^, Lei Zhai^3^, Xuejian Yu^3^, Ying Li^4^, Pengfei Zhu^1,4^, Rongze Chen^1,5,6,7^, Xiaoyan Jing^1,5,6,7^, Gongchao Jing^1,5,6,7^, Shiqi Zhou^1,5,6^, Mingyue Xu^1,5,6^, Chen Wang^1,5,6^, Changkai Niu^2^, Yuanyuan Ge^3^, Bo Ma^1,5,6,7^, Gaishuang Shang^2^, Yunlong Cui^2,*^, Su Yao^3,*^, Jian Xu^1,5,6,7,*^

^1^Single-Cell Center, CAS Key Laboratory of Biofuels, Shandong Key Laboratory of Energy Genetics, Qingdao Institute of Bioenergy and Bioprocess Technology, Chinese Academy of Sciences, Qingdao, Shandong, China

^2^Eastsea Pharma Co., Ltd, Qingdao, Shandong, China

^3^China National Research Institute of Food and Fermentation Industries Co., LTD., China Center of Industrial Culture Collection, Beijing, China

^4^Qingdao Single-Cell Biotech. Co., Ltd, Qingdao, Shandong, China

^5^Shandong Energy Institute, Qingdao, Shandong, China

^6^Qingdao New Energy Shandong Laboratory, Qingdao, Shandong, China

^7^University of Chinese Academy of Sciences, Beijing, China

^8^College of Information Science & Engineering, Ocean University of China, Qingdao, Shandong, China

^9^Qingdao Branch of China United Network Communications Co., Ltd, Qingdao, Shandong, China

^*^Correspondence to: [xujian@qibebt.ac.cn](mailto:xujian@qibebt.ac.cn) (Jian Xu), [su.yao@china-cicc.org](mailto:su.yao@china-cicc.org) (Su Yao) and [cuiyunlong@qdeastsea.cn](mailto:cuiyunlong@qdeastsea.cn) (Yunlong Cui)

Jia Zhang, Lihui Ren, Lei Zhang, and Yanhai Gong contributed equally to this study.

**Contents**

- **Supplementary Methods**
- **Supplementary Tables and Figures**

Table S1. Commercial probiotic products used for evaluating the SCIVVS method in this study.

Table S2. Viable-cell counting of Product X and Product Y using the traditional plate-counting method or the SCIVVS method.

Table S3. The reference dataset of Single-cell Raman Spectra (SCRS) from 21 standard probiotic strains that represent the standard statutory strains for human consumption.

Table S4. Determination of species composition and proportion in MPP-A based on Single-cell Raman Spectra, 16S-rDNA-based amplicon sequencing, or whole-metagenome sequencing.

Table S5. Source tracking of single bacterial cells directly from commercial probiotic products based on one-cell 16S rDNA amplicon sequencing via scRACS-Seq.

Table S6. The reference dataset of Single-cell Raman Spectra from five probiotic strains in CPP-A.

Table S7. The live-cell counts and proportions of each strain in CPP-A.

Table S8. Cost evaluations and technical procedure for a comprehensive quality assessment process of probiotic products using the SCIVVS method.

Figure S1. The workflow for determining “Metabolic Activity Level” (MAL) as the basis for the vitality test.

Figure S2. The number of colonies on culture plates for the MPP-A product incubated with 100% D2O, either for 3 hours or for 0 hours.

Figure S3. The microscopic view of probiotic cells for automated cell counting at the first step of SCIVVS.

Figure S4. Agarose gel electrophoresis of MDA products or 16S PCR products that were amplified from the probiotic cells sorted by RACS.

Figure S5. Intensity of deuterium incorporation in 100% D2O MRS media of CPP-A at single-cell resolution.

Figure S6. The phylogenetic tree reconstructed using the SNPs of all the individually sequenced SAGs of *L. plantarum* that are of varying genome-wide coverage.

- **Supplementary references**

**Supplementary Methods**

***Automated acquisition by scRACS-Seq*** All SCRS were acquired on a RACS-Seq instrument (Qingdao Single-cell Biotechnology, Qingdao, China) as described previously [1]. The system carries a microscope with 100 × dry objective (NA = 0.80) and a 532 nm Nd: YAG laser with a maximum power of 50 mW. Each cell was exposed to laser for 2 s and spectra were recorded with 300 grooves/mm diffraction grating.

Automated cell counting was performed in two steps: single-cell segmentation and localization, and automated SCRS-based live-cell counting. Firstly, we developed a fully automated high-throughput solution named DCN, which can achieve the segmentation of probiotic cells. In DCN, each pixel is classified into a category and an output scoring matrix is generated, which is the probability that each pixel in the image belongs to each category. Finally, based on this output matrix, each pixel can be classified and a segmentation result can be generated. As an improved network based on FCN, DCN can enlarge the receptive field of network without changing the calculation points or lowering image resolution [2,3]. Under the same convolution kernel, dilated convolution retains richer image information than the conventional convolution. In this work, the dilated convolution was placed at the 2^nd^ and 4^th^ layers of network to better express the cell picture.

Several indicators were used to evaluate the performance of segmentation methods: accuracy (ACC), intersection-over-union (IoU), false positive rate (FPR), false negative rate (FNR) and overall error rate (OER). ACC represents the pixel removal accuracy in each group; IoU is the overlapped percentage between the predicted and target output; FPR is the error rate indicating that the background pixel was wrongly included in target objects; FNR is the false identification rate that the target pixel is assigned as background; OER is the proportion of misidentified pixels. These parameters are described as Eqs. (1)-(5):

$\mathrm{ACC}=\frac{TP+TN}{TP+FP+TN+FN}$

(1)

(2)

(3)

(4)

(5)

$$IoU=\frac{TP}{TP+FP+FN}$$

$$FPR=\frac{FP}{FP+TN}$$

$$FNR=\frac{FN}{FN+TP}$$

$$OER=\frac{FN+FP}{TP}$$

Where TP, TN, FP and FN represent the number of pixels for true positive, true negative, false positive and false negative respectively. Subsequently, the system automatically initiates the spectrum acquisition process and then quality assessment and screening, to obtain high-quality Raman spectra [4].

***Automated live-cell counting in the RACS-Seq instrument for probiotic products*** The commercial probiotic product was suspended in 0.85% sterile physiological saline (NaCl) and diluted with a dilution gradient of 10 times. Then it was suspended with 1 mL 100% D_2_O MRS medium and incubated for 3 hrs. After washing three times with deionized water to remove the medium, we adjusted the cell density and added Tween 20 with a final concentration of 0.2% to ensure cell dispersion in the field of vision. Then 1 μL cell suspension was transferred to calcium fluoride glass slide (CaF_2_) and five visual fields (visual fields in four corners and middle; total 660 visual fields in 1 μL cell suspension) were photographed under 100 x microscope. All the SCRS of sample in the five visual fields were collected (CNVF: cells number in the five visual fields) and the background spectrum was collected for background subtraction of samples. Thus, the cell number for product was calculated as $\frac{CNVF}{5}$×660×dilution times.

***Cell counting for probiotics products by the conventional methods*** A hemocytometer chamber was typically used to calculate the total number of cells in commercial probiotic products. After 1:1000 dilution of probiotic products, a drop of the preparation samples was placed in the chamber, and cells in four corners and middle squares were counted. Then the values were multiplied by the numbers of squares and the dilution factor to obtain the total number of cells (including both live and dead). On the other hand, the plate-colony counting method was used to calculate the number of live cells. The probiotic product was diluted 10-fold in series and cultured on the MRS solid plate. Three plates were used for each sample as biological duplication, and then cultured under anaerobic conditions at 37℃ for 72 hrs. The average number of colonies on plates was recorded and multiplied by the dilution factor to obtain the total number of live cells.

***Metabolic activity quantification via RACS-Seq for probiotic products*** The intake of H_2_O or D_2_O is a fundamental feature of living cells. In the presence of D_2_O, the electron carriers NADPH and NADH related to cell metabolic activity can exchange H^+^ in NADPH+H^+^ with D^+^ from D_2_O, and incorporate D^+^ to form C-D bonds. Thus, the metabolic vitality of a cell can be distinguished by the C-D bond from 2040 to 2300 cm^−1^ [5]. The C-D ratios (CDR) was defined as percentage of the integrated spectral intensity of the C-D band (2040-2300 cm^−1^) as compared to the sum of the C-D band and the predominant C-H band (2800-3100 cm^−1^), so as to quantify the degree of D substitution in C-H bonds [5]. Thus, we introduced the concept of “Metabolic Activity Level” (MAL), which corresponds to the extent of cellular vitality of probiotic products: $MAL={CDR}_{sample}-{CDR}_{0h}$.Especially, the CDR*_sample_* and CDR*_0h_* are the average CDR value of probiotic products incubated with 100% D_2_O for 3 hrs or 0 hrs, respectively. A higher MAL represents higher vitality (MAL ≤ 0, dead cells; MAL > 0, living cells). All the MAL values were automatically calculated by R software through a script. Additionally, we introduced the concept of “relative Metabolic Activity Level” (rMAL), which corresponds to the relative vitality of a cell: $rMAL=\frac{{CDR}_{sample}-{CDR}_{0h}}{{CDR}_{control}-{CDR}_{0h}}$. Especially, the CDR*_control_* is the average CDR value of a pure-culture strain in its logarithmic growth period (12 h) incubated with 100% D_2_O for 3 hrs. The mean rMAL would range from 0 to 1, with higher rMAL value representing higher vitality retained.

***Method for goodness-of-fit test*** Goodness-of-fit test is a statistical hypothesis test for the consistency between the observation data and the theoretical data calculated according to a certain hypothesis or distribution model, so as to judge whether the hypothesis or model is consistent with the actual observation. The observed values were divided into *k* groups according to the number of probiotic species in the product. The observation frequency of each group of *n* times of observations was recorded as *Oi*. Based on the distribution law of variables or the probability algorithm, the theoretical frequency of each group was calculated as *Pi*, and the theoretical frequency of each group as Ti. The significance of difference between *Oi* and *Ti* was tested, and the inconformity between them judged. Null hypothesis H0: O-T = 0; alternative hypothesis H1: O-T ≠ 0 (the test here is to judge whether the observation number conforms to the theoretical distribution). The rejection field was established according to $\chi^{2}=\sum_{i=1}^{k} \frac{{(O_{i}-T_{i})}^{2}}{T_{i}}$ to draw a statistical conclusion.

***Construction of a SCRS reference database for 21*** ***standard statutory strains of probiotics*** The 21 strains, from three genera including *Lactobacillus*, *Bifidobacterium* and *Streptococcus* were stored at -80 °C, and resuscitated on MRS or BS agar plate about 48 - 72 h prior to the experiment. Single clones were then cultured in MRS or BS broth at 37 °C for 3 hrs under microaerobic, aerobic and anaerobic conditions, respectively. The medium was removed and the cells were washed three times with deionized water. The cell density was adjusted to ensure that each cell was fully dispersed on the slide. After washing and re-suspension, 1 μL cell suspension was transferred to calcium slide (CaF_2_).

***Bacterial strain classification using SCRS*** The CNN architecture was adapted from Resnet-18 which is widely successful across a range of computer vision tasks. It consists of an initial convolution layer followed by four residual layers and a final fully connected classification layer (a block diagram in **Figure 4B**). The residual layers contain shortcut connections between the input and output of each residual block, allowing for better gradient propagation and stable training. Each residual layer contains four convolutional layers, thus the total depth of the network is 18 layers. The initial convolution layer has 64 convolutional filters, while each of the four convolutional layers has 100 filters. These architecture hyperparameters were selected via grid search using one training and validation split on the classification task.

We first trained the CNN model on the 21 cultured strains classification task, where the output of the CNN is a vector of probabilities across the 21 classes and the maximum probability is taken as the predicted class. We used the Adam optimizer across all experiments with learning rate 0.001, betas (0.5, 0.999), and batch size 2. Classification accuracies were reported across randomly selected train and validation splits. For the split, we split the total cultured data into 80/20 training and validation splits, trained the CNN on the training split, and used the accuracy on the validation split to perform model selection. In the training stage of CNN model, the input data was divided into two parts: spectral data (matrix m*593, m is the number of spectra, 593 is the spectrum dimension, called X), category label (matrix m*1, m is the number of spectra, 1 is the corresponding category, called Y). The algorithm would build a mapping relationship between X and Y. In the model prediction stage, the input data was only spectral data (n*593 matrix), and the category label would be predicted. We then evaluated and reported the test accuracy on the test dataset which was gathered from independently cultured and prepared samples. We used the same data for three times of modeling and verification, and the accuracy results were based on the average and deviation value of 3 repeated modeling. For isolates and mock clinical samples, we used the model to determine whether they are *L. plantarum* and the probability.

***Metagenomic profiling of commercial probiotic products*** For the 16S sequencing data, paired-end reads were firstly merged using FLASH v1.2.7 [6]; then, merged reads were clustered into OTUs using Qiime 1.9.1 [7]; finally, OTUs were taxonomically annotated based on Silva database [8]. For the shotgun metagenomic sequencing data, low quality and adapter sequences were filtered; then, clean reads were taxonomically annotated and quantified using Kraken2 [9,10].

***Sorting of target probiotic cell directly from a probiotic product by scRACS-Seq*** Before conducting the scRACS-Seq experiment, all reagents and consumable materials were ultraviolet irradiated for 10 min in Spectrolinker XL-1500 in order to remove pollution. Then, the commercial probiotic product was dissolved in 0.85% sterile physiological saline (NaCl) and shaken for 5-10 minutes to dissolve completely. After centrifugation at 2000 g for 3 minutes, supernatant was removed and the cells in suspension were washed three times with ddH_2_O and diluted to a proper density (≈10^6^ CFU/mL). Then, the cellular sample was injected into a RACS-Seq chip on the height adjustable sample holder for SCRS acquisition and sorting. After loading the chip onto the RACS-Seq instrument (Qingdao Single-cell Biotech, Qingdao, China), a 10x dry objective was used for observation of droplet generation and transportation, while a 60x water objective was used for both SCRS acquisition and optical tweezers. The cells in the detection fields were captured, either without or with SCRS acquisition (the 532 nm laser for 3 s; the C-D ratios used for identifying live cell). Thus, SCRS-based phenotypes and subsequent genotypes were linked in an accurately indexed manner.

***Multiple displacement amplification*** ***(MDA) and sequencing of RACS-sorted probiotic cell*** Before conducting MDA test, all reagents and consumable materials were ultraviolet irradiated for 10 min in Spectrolinker XL-1500. Then the single sorted cell in the PCR tube was lysed with 1 µL Lysis buffer with transient vortex at 65℃ for 10 min, followed by addition of 1 µL stop solution (B buffer) to neutralize the lysis buffer. After gently shaking, 30 µL reagents containing 6N primers, dNTPs, DTT and phi29 DNA polymerase were added and incubated at 30 ℃ for 8 hrs (T100, Bio-Rad, California, USA). Negative control reactions without cells or DNA template were carried out in parallel, to detect and quantify the potential contamination from laboratory environment. All reagents were from the Single-cell Whole-genome Amplification Kit provided by Qingdao Single-Cell Biotech. Ltd.

For quality assessment of the MDA products, 16S rRNA gene sequencing and shotgun sequencing were both tested. The 16S PCR reaction mix contains 10 µL 2×Taq Mix (Monad Biotech Co., Ltd.), 1 µL 27F primer (10 µM), 1 µL 1492R primer (10 µM), 7 µL nuclease-free water and 1 µL MDA products. The 16S PCR program: 94 °C for 5 min, 30 cycles of 94 °C for 30 s, 57 °C for 30 s, 72 °C for 1 min and 72 °C for 10 min. The MDA products, after passing quality control, would proceed for library construction for shotgun sequencing (Novogene Beijing, China; Illumina PE150 sequencing platform).

***Analyses of post-RACS one-probiotic-cell genome sequencing*** The main computational pipeline (<https://github.com/gongyh/nf-core-scgs>) to analyze the obtained one-cell genome sequencing datasets was as previously described [11]. Briefly, raw reads from each SAG were quality trimmed using Trim Galore (https://www.bioinformatics.babraham.ac.uk/projects/trim_galore/) in paired-end mode. To detect contaminated DNA fragments, clean reads were phylogenetically classified using Kraken [12]. Clean reads were then assembled into contigs using SPAdes [13] in single-cell mode. Taxonomic composition of assembled contigs (longer than 200 bp) was visualized using BlobTools [14]. Assembled genomes were annotated using Prokka [15]. Genome completeness and the degree of contamination for the assembled contigs were estimated using CheckM [16]. When necessary, assembled contigs were further split into bins by taxonomic annotations (in the genus level) for each SAG.

To perform source tracking based on the SAGs, external reference genomes obtained from pure-cultured isolates were obtained from NCBI RefSeq database (atypical genomes were excluded). Then, potentially misclassified genomes with ANI values < 97% (as compared to our reference genomes) were also excluded. ANI values were calculated using OAT [17]. In the end, 283 genomes were obtained for *L. paracasei*, and 744 genomes for *L. plantarum*. Core-genome SNPs were calculated using Parsnp [18]. The phylogenetic trees were drawn using the R package ggtree and MEGA software [19,20].

**Supplementary Tables and Figures**

**Table S1. Commercial probiotic products used for evaluating the SCIVVS method in this study.**

| **Name cited** | **Manufacturer** | **Type** | **Strains in product** |
| --- | --- | --- | --- |
| MPP-A | Renhe Group^®^ | Single-strain | *L. plantarum* 299V |
| Product X | Eastsea Pharma^®^ | Single-strain | *B. infantis* (produced without microencapsulation) |
| Product Y | Eastsea Pharma^®^ | Single-strain | *B. infantis* (produced with microencapsulation) |
| CPP-A | Eastsea Pharma^®^ | Multi-strain | *L. plantarum, L. paracasei, B. coagulans, B. animalis,* *L. rhamnosus* |

**Table S2. Viable-cell counting of Product X and Product Y** **using the traditional plate-counting method or the SCIVVS method.** Before and after storage at 37℃ for 10 days (Accelerated Experiment), viable-cell number and survival rate for Product X and Product Y were calculated. Acceleration experiment is used to predict the storage potential of the products produced by different processes through short-term (several days) and high temperature treatment simulations. Before Acceleration Experiment, viable-cell number for Product X and Product Y were calculated by SCIVVS method.

|  | | **Product X** | **Product Y** |
| --- | --- | --- | --- |
| **Viable-cell number before acceleration experiment** | **Plate count (CFU/g)** | 3.40 ± 0.36 × 10^9^ | 2.51 ± 0.06 × 10^9^ |
|  | **SCIVVS (CFU/g)** | 3.43 ± 0.18 × 10^9^ | 2.65 ± 0.18 × 10^9^ |
|  | **P value** | H_0_: *p* > 0.05 | H_0_: *p* > 0.05 |
| **Viable-cell number**  **after acceleration experiment** | **Plate count (CFU/g)** | 3.57 ± 0.25 × 10^7^ | 2.08 ± 0.14 × 10^9^ |
|  | **Survival rate** | 1.05% | 82.87% |

**Table S3. The reference dataset of Single-cell Raman Spectra (SCRS) from 21 standard probiotic strains that represent the standard statutory strains for human consumption.** To build a training dataset of SCRS for probiotic identification, for a total of 21 standard probiotic strains, we deposited probiotic bacterial cells onto a CaF_2_ slide and collected a training test of 3546 spectra and a test dataset of 1519 spectra.

| **Organisms** | | **Type Strain** | **Training-spectra Number** | **Test-spectra Number** |
| --- | --- | --- | --- | --- |
| **Original name** | **Updated name** |  |  |  |
| *Lactobacillus delbrueckii* subsp. *lactis* | No change | DSM20072 | 147 | 63 |
| *Lactobacillus crispatus* | No change | CICC 24879 | 168 | 72 |
| *Lactobacillus johnsonii* | No change | CICC 6252 | 168 | 72 |
| *Lactobacillus salivarius* | *Ligilactobacillus salivarius* | DSM20555 | 165 | 71 |
| *Bifidobacterium breve* | No change | CICC 6079 | 192 | 82 |
| *Bifidobacterium adolescentis* | No change | CICC 6070 | 172 | 74 |
| *Bifidobacterium animalis* | *Bifidobacterium animalis* subsp. *animalis* | CICC 6250 | 187 | 80 |
| *Lactobacillus casei* | *Lacticaseibacillus casei* | CICC 6117 | 176 | 76 |
| *Lactobacillus rhamnosus* | *Lacticaseibacillus rhamnosus* | CICC 6001 | 193 | 83 |
| *Lactobacillus helveticus* | No change | CICC 6032 | 164 | 70 |
| *Lactobacillus bulgaricus* | *Lactobacillus delbrueckii* subsp. *bulgaricus* | CICC 6047 | 177 | 75 |
| *Lactobacillus reuteri* | *Limosilactobacillus reuteri* | CICC 6226 | 161 | 69 |
| *Lactobacillus paracasei* | *Lacticaseibacillus paracasei* | CICC 6228 | 165 | 70 |
| *Lactobacillus fermentum* | *Limosilactobacillus fermentum* | CICC 24209 | 155 | 66 |
| *Lactobacillus gasseri* | No change | CICC 24878 | 191 | 82 |
| *Bifidobacterium infantis* | *Bifidobacterium longum* subsp. *infantis* | BNCC 341709 | 137 | 59 |
| *Lactobacillus plantarum* | *Lactiplantibacillus plantarum* | CICC 6009 and  DSM 9843 | 237 | 102 |
| *Streptococcus thermophilus* | No change | CICC 6063 | 139 | 60 |
| *Bifidobacterium longum* | *Bifidobacterium longum* subsp. *longum* | CICC 6069 | 146 | 62 |
| *Bifidobacterium bifidum* | No change | CICC 6071 | 189 | 81 |
| *Lactobacillus acidophilus* | No change | CICC 6074 | 117 | 50 |

**Table S4. Determination of species composition and proportion in MPP-A based on Single-cell Raman Spectra, 16S-rDNA-based amplicon sequencing, or whole-metagenome sequencing.** Abundance of strains in MPP-A calculated by different methods are listed in parathesis.

| **Sample** | **16S-rDNA amplicon sequencing based ID**  **(Relative abundance)** | **SCRS-based ID (Predicted abundance)** | **Metagenome sequencing**  **based ID**  **(Relative abundance)** |
| --- | --- | --- | --- |
| MPP-A | *L. plantarum* (83.04%) | *L. plantarum* (92.72%) | *L. plantarum* (98.28%) |

**Table S5. Source tracking of single bacterial cells directly from commercial probiotic products based on one-cell 16S rDNA amplicon sequencing via scRACS-Seq.**

| **Sample** | **Strains** | **Percent identity** |
| --- | --- | --- |
| A1 | *L. rhamnosus* | 99.73% |
| A2 | *L. rhamnosus* | 99.66% |
| A3 | *L. rhamnosus* | 99.72% |
| A4 | *L. rhamnosus* | 99.79% |
| B1 | *L. rhamnosus* | 99.73% |
| B3 | *L. plantarum* | 99.55% |
| B4 | *L. paracasei* | 99.40% |
| B6 | *L. plantarum* | 99.52% |
| B7 | *L. paracasei* | 99.72% |
| B8 | *L. rhamnosus* | 99.73% |
| B9 | *L. paracasei* | 99.72% |
| B10 | *L. plantarum* | 99.66% |
| B11 | *L. rhamnosus* | 99.86% |
| B13 | *L. rhamnosus* | 99.73% |
| C1 | *L. paracasei* | 99.80% |
| C2 | *L. paracasei* | 99.86% |
| C3 | *L. plantarum* | 99.59% |
| C4 | *L. paracasei* | 99.86% |
| C5 | *L. paracasei* | 99.57% |
| C6 | *L. plantarum* | 99.11% |
| C7 | *L. plantarum* | 99.59% |
| C8 | *L. paracasei* | 99.80% |
| C9 | *L. plantarum* | 99.59% |
| C10 | *L. paracasei* | 99.59% |
| C11 | *L. plantarum* | 99.86% |
| C12 | *L. paracasei* | 99.86% |
| C13 | *L. plantarum* | 99.52% |
| C14 | *L. paracasei* | 99.79% |
| C15 | *L. plantarum* | 99.79% |
| C16 | *L. paracasei* | 99.86% |

**Table S6. The reference dataset of Single-cell Raman Spectra from five probiotic strains in CPP-A.** To build a model for SCRS-based identification, for five bacterial strains, we collected a training test of 1602 spectra and a test dataset of 401 spectra.

| **Type strain** | **Num. of training-spectra** | **Num. of test-spectra** |
| --- | --- | --- |
| *B. animalis* | 347 | 75 |
| *L. paracasei* | 326 | 79 |
| *B. Coagulans* | 320 | 67 |
| *L. rhamnosus* | 294 | 97 |
| *L. plantarum* | 315 | 83 |

**Table S7. The live-cell counts and proportions of each strain in CPP-A.** Five strains in CPP-A were mixed according to a certain proportion of live cells to obtain the "actual proportion". Then, the live cells proportion and the live cells number of five strains in CPP-A were derived by SCIVVS.

| **Strains** | **Live-cell proportion** | | **Live-cell number**  **SCIVVS** |
| --- | --- | --- | --- |
|  | **Actual proportion** | **SCIVVS** |  |
| *B. animalis* | 17.08% | 15.66 ± 0.01% | 6.01 ± 0.05 × 10^10^ CFU/g |
| *L. paracasei* | 54.10% | 54.22 ± 0.03% | 2.08 ± 0.10 × 10^11^ CFU/g |
| *B. coagulans* | 2.02% | 2.28 ± 0.01% | 8.76 ± 0.03 × 10^9^ CFU/g |
| *L. rhamnosus* | 0.56% | 1.05 ± 0.01% | 4.03 ± 0.02 × 10^9^ CFU/g |
| *L. plantarum* | 26.24% | 26.79 ± 0.01% | 1.03 ± 0.04 × 10^11^ CFU/g |

**Table S8. Cost evaluations and technical procedure for a comprehensive quality assessment process of probiotic products using the SCIVVS method.**

| **Cost evaluations** | | **Technical procedures** | |
| --- | --- | --- | --- |
| **Reagent** | **Cost** | **Process** | **Duration** |
| 100% D_2_O MRS (1 mL) | $0.71 | Pretreatment | 0.5 hrs |
| 0.22 μm PES membrane filter (1 PCS) | $1.11 | D_2_O incubation | 3 hrs |
| AnaeroPack (1 PCS) | $2.36 | Chip preparation | 0.5 hrs |
| Tubes (2 PCS; 1.5 mL and 15 mL) | $0.21 | SCRS acquired | 1 hrs |

**Figure S1. The workflow for determining “Metabolic Activity Level” (MAL) as the basis for the vitality test.** MAL corresponds to the extent of cellular vitality of probiotic products: $MAL={CDR}_{sample}-{CDR}_{0h}$.Especially, the CDR*_sample_* and CDR*_0h_* are the average CDR value of probiotic products incubated with 100% D_2_O for 3 hours or 0 hours, respectively. A higher MAL would represent higher vitality (MAL ≤ 0, dead cells; MAL > 0, live cells).

**
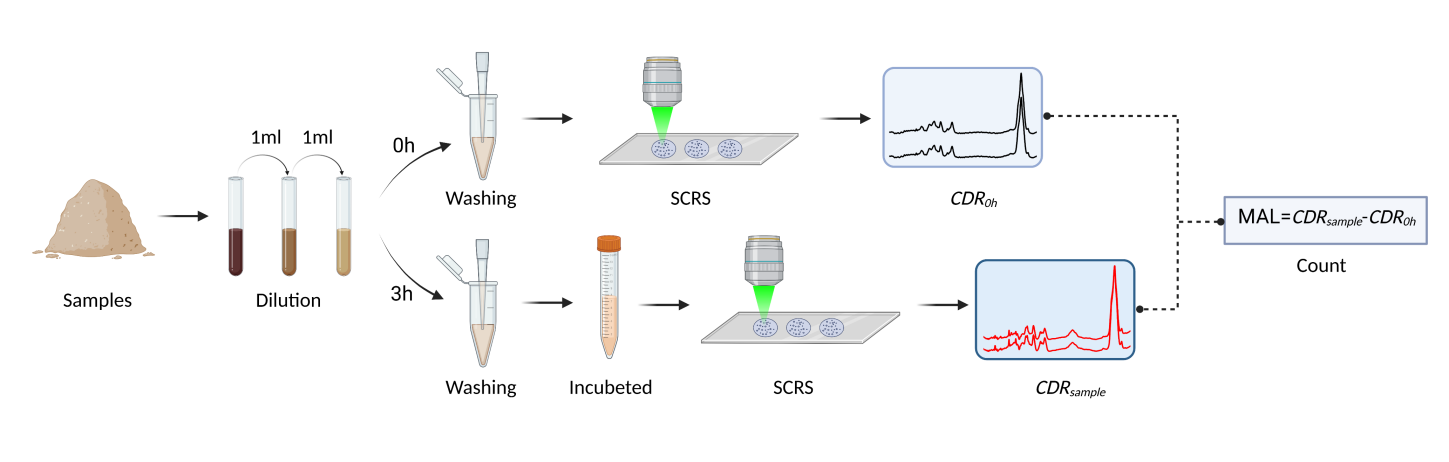
**

**Figure S2. The number of colonies on culture plates for the MPP-A product incubated with 100% D_2_O, either for 3 hours or for 0 hours.** A, B and C are the three batches of MPP-A, respectively. Each sample was analyzed in triplicate.


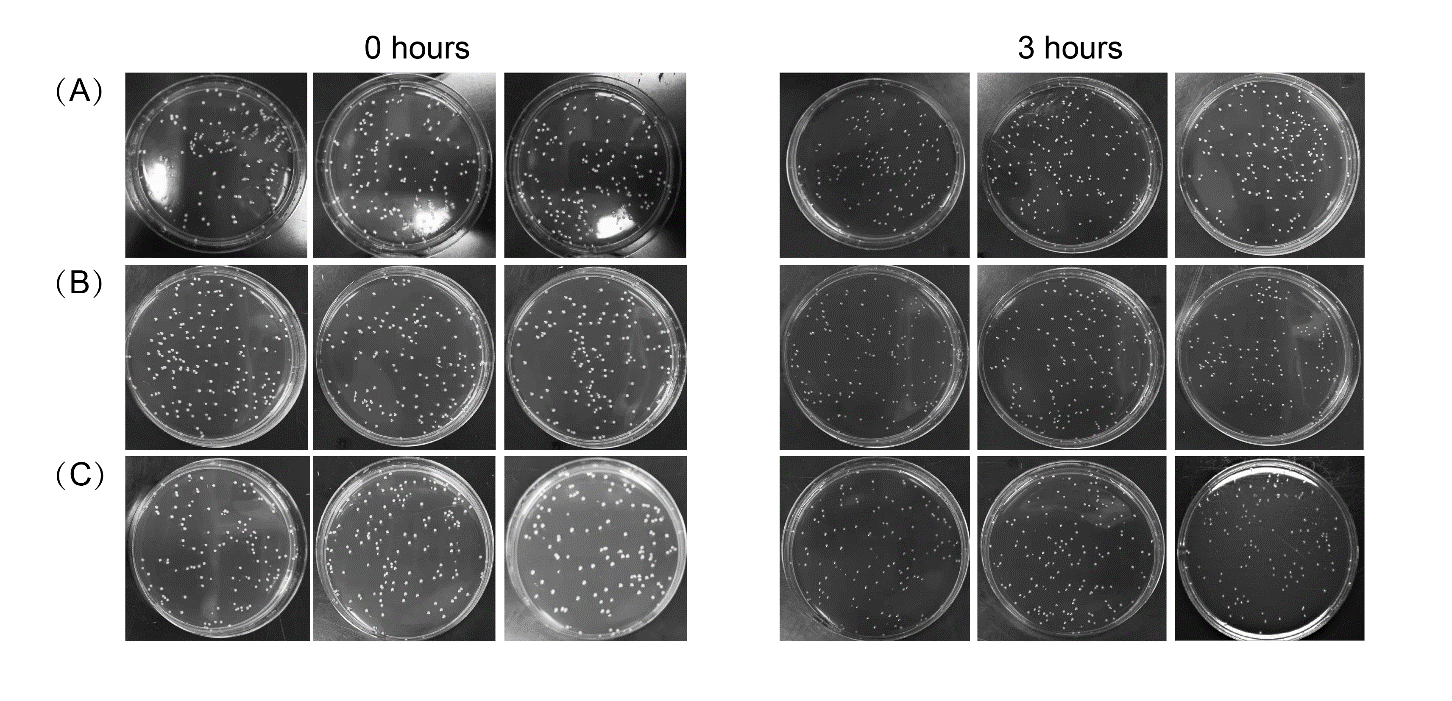


**Figure S3. The microscopic view of probiotic cells for automated cell counting at the first step of SCIVVS.** Three batches (**A**, **B** and **C**) are shown for the MPP-A product (*Lactobacterium plantarium*), respectively. Each sample was incubated with 100% D_2_O for 3 hours before microscopic analysis in a RACS-Seq instrument. Five visual fields of each sample were analyzed automatically.

**
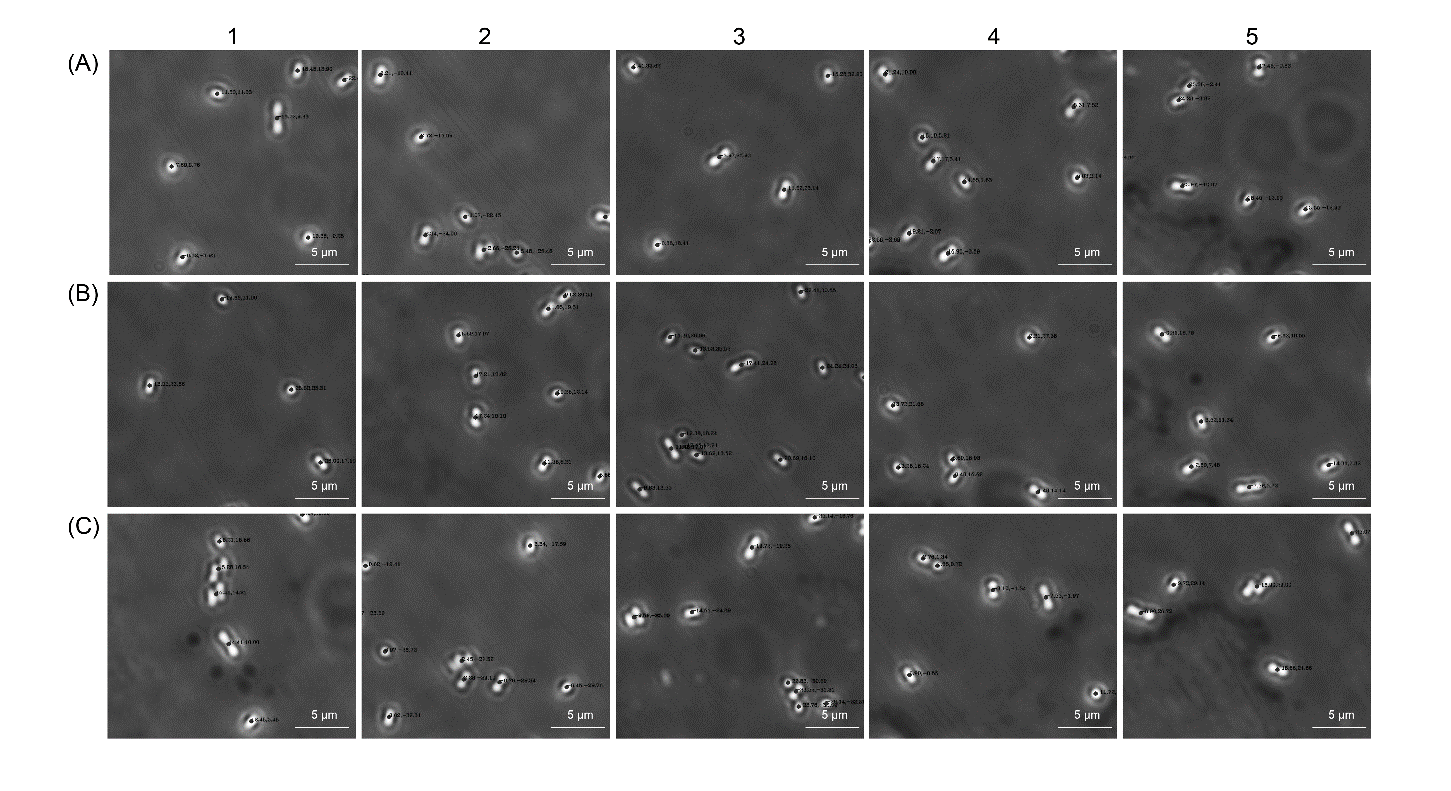
**

**Figure S4. Agarose gel electrophoresis of MDA products or 16S PCR products that were amplified from the probiotic cells sorted by RACS.** (**A**) Lanes A1, A2, A3, A4: the sorted cells of *L. plantarum* from MPP-A. Lanes O5: the sorted empty droplet (without any cell) as negative control. (**B**) Lanes B1, B2, B3, B4, B5, B6, B7, B8, B9, B10, B11, B12, B13: the sorted cells from a mock microbiota (*L. paracasei* vs *L. rhamnosus* vs *L. plantarum*; 1:1:1); Lanes O14: the sorted empty droplet (without any cell) as negative control.


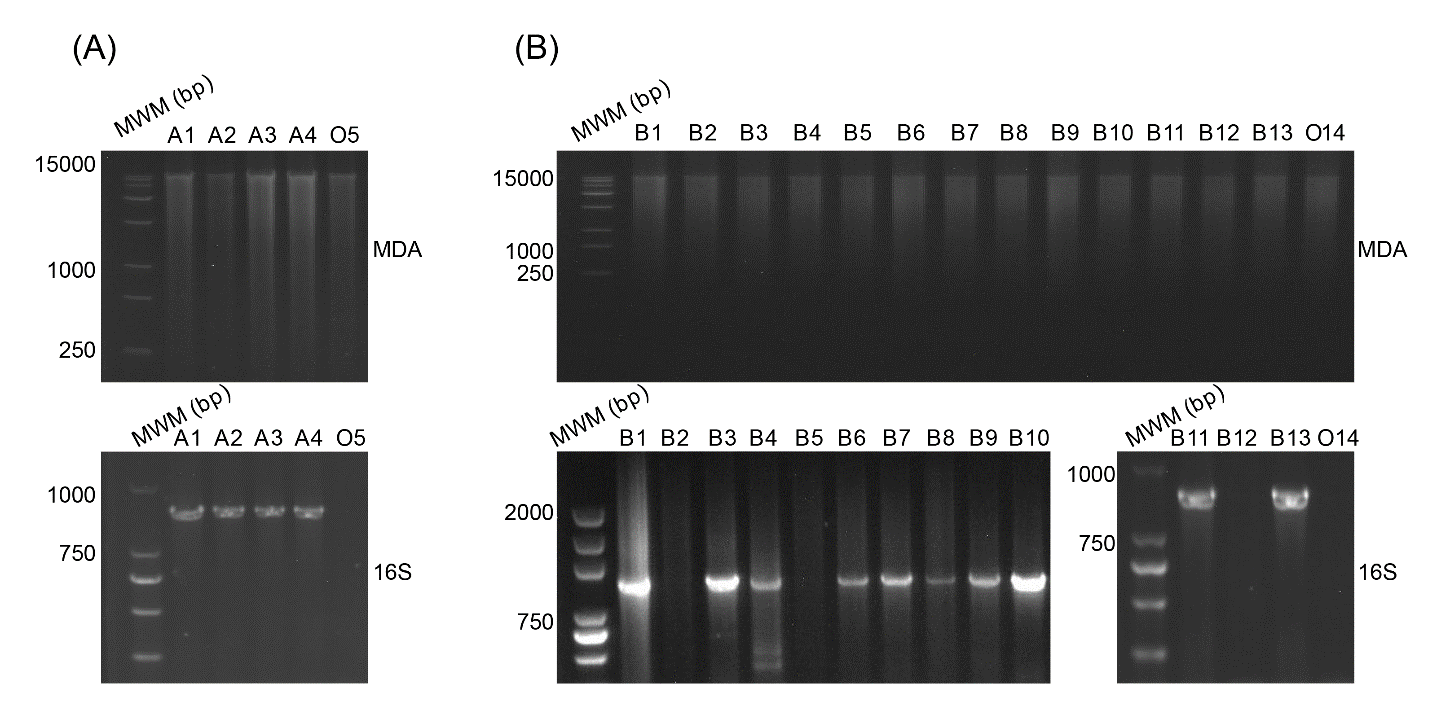


**Figure S5. Intensity of deuterium incorporation in 100% D_2_O MRS media of CPP-A at single-cell resolution.** The percentages of active cells were shown for those samples incubated for 0, 1, 2, 2.5, 3, 3.5 and 4 hrs.


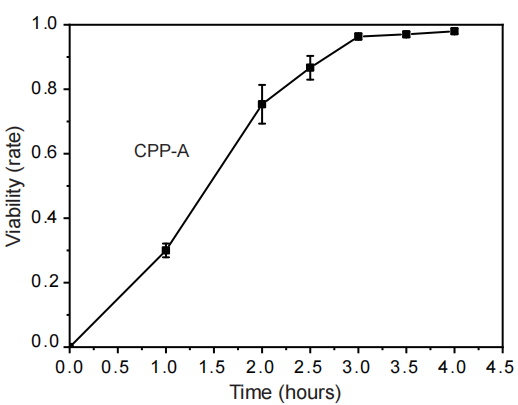


**Figure S6.** **The phylogenetic tree reconstructed using the SNPs of all the individually sequenced SAGs of *L. plantarum* that are of varying genome-wide coverage.** The bootstrap confidence values (as percentages) are indicated above each branch. The scale bar represents a Jukes-Cantor distance of 0.0005. Single cells (all from this study) are indicated by black circles; bulk-culture based sequencing results are indicated by black triangles.


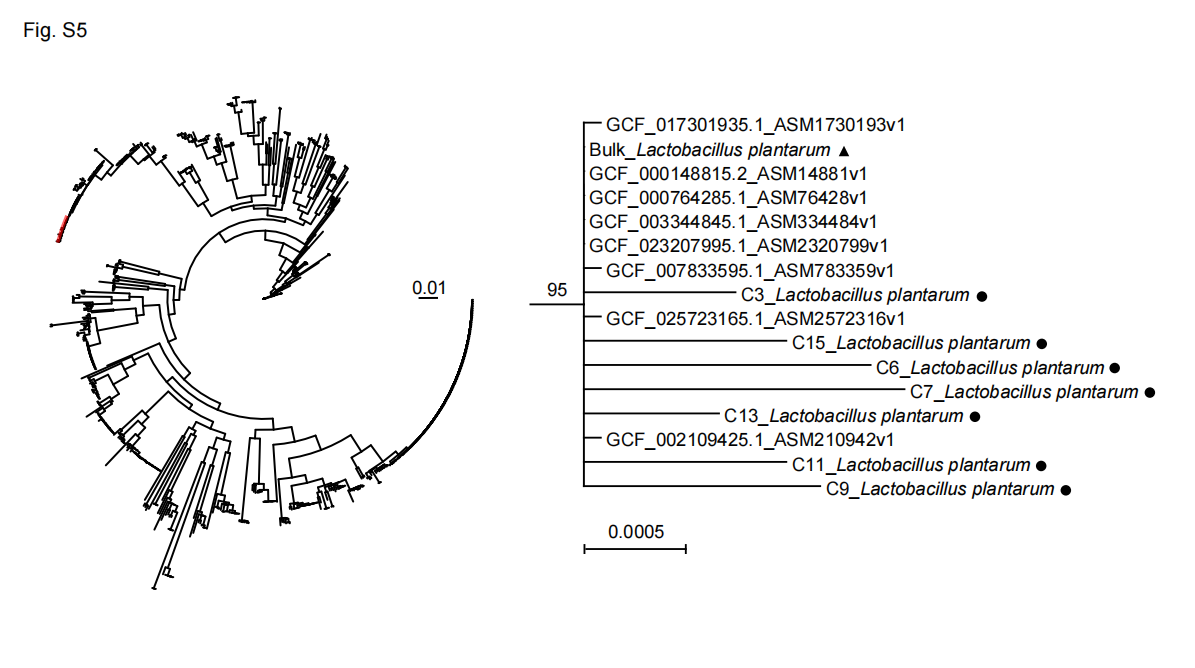


**Supplementary references**

1. Xu, Teng., Yanhai. Gong, Xiaolu. Su, Pengfei. Zhu, Jing. Dai, Jian. Xu, and Bo. Ma. 2020. "Phenome-Genome Profiling of Single Bacterial Cell by Raman-Activated Gravity-Driven Encapsulation and Sequencing." *Small* 16(30): e2001172. <https://doi.org/10.1002/smll.202001172>

2. Zhang, Meng., Weili. Hong, Nader. S. Abutaleb, Junjie. Li, Pu. Ting. Dong, Cheng. Zong, Pu. Wang, et al. 2020. "Rapid Determination of Antimicrobial Susceptibility by Stimulated Raman Scattering Imaging of D(2)O Metabolic Incorporation in a Single Bacterium." *Advanced Science (Weinh)* 7(19): 2001452. <https://doi.org/10.1002/advs.202001452>

3. Bauer, D., K. Wieland, L. Qiu, A. C. Neumann-Cip, G. Magistro, C. Stief, A. Wieser, et al. 2020. "Heteroresistant Bacteria Detected by an Extended Raman-Based Antibiotic Susceptibility Test." *Analytical Chemistry* 92(13): 8722-31. <https://doi.org/10.1021/acs.analchem.9b05387>

4. Zhu, Pengfei, Lihui Ren, Ying Zhu, Jing Dai, Huijie Liu, Yuli Mao, Yuandong Li, et al. 2022. "Rapid, automated, and reliable antimicrobial susceptibility test from positive blood culture by CAST‐R." *mLife* 1: 329-40. <https://doi.org/10.1002/mlf2.12019>

5. Tao, Yifan, Yun Wang, Shi Huang, Pengfei Zhu, Wei E. Huang, Junqi Ling, and Jian Xu. 2017. "Metabolic-Activity-Based Assessment of Antimicrobial Effects by D2O-Labeled Single-Cell Raman Microspectroscopy." *Analytical Chemistry* 89(7): 4108-15. <https://doi.org/10.1021/acs.analchem.6b05051>

6. Magoč, Tanja, and Steven L. Salzberg. 2011. "FLASH: fast length adjustment of short reads to improve genome assemblies." *Bioinformatics* 27(21): 2957-63. <https://doi.org/10.1093/bioinformatics/btr507>

7. Caporaso, J. Gregory., Justin. Kuczynski, Jesse. Stombaugh, Kyle. Bittinger, Frederic. D. Bushman, Elizabeth. K. Costello, Noah. Fierer, et al. 2010. "QIIME allows analysis of high-throughput community sequencing data." *Nature methods* 7(5): 335-6. <https://doi.org/10.1038/nmeth.f.303>

8. Quast, Christian, Elmar Pruesse, Pelin Yilmaz, Jan Gerken, Timmy Schweer, Pablo Yarza, Jörg Peplies, et al. 2013. "The SILVA ribosomal RNA gene database project: improved data processing and web-based tools." *Nucleic Acids Research* 41(D1): D590-D96. <https://doi.org/10.1093/nar/gks1219>

9. Wood, Derrick. E., Jennifer. Lu, and Ben. Langmead. 2019. "Improved metagenomic analysis with Kraken 2." *Genome Biology* 20(1): 257. <https://doi.org/10.1186/s13059-019-1891-0>

10. Lu, Jennifer., Natalia. Rincon, Derrick. E. Wood, Florian. P. Breitwieser, Christopher. Pockrandt, Ben. Langmead, Steven. L. Salzberg, et al. 2022. "Metagenome analysis using the Kraken software suite." *Nature Protocols* 17(12): 2815-39. <https://doi.org/10.1038/s41596-022-00738-y>

11. Jing, Xiaoyan, Yanhai Gong, Huihui Pan, Yu Meng, Yishang Ren, Zhidian Diao, Runzhi Mu, et al. 2022. "Single-cell Raman-activated sorting and cultivation (scRACS-Culture) for assessing and mining in situ phosphate-solubilizing microbes from nature." *isme communications* 2(1): 106. <https://doi.org/10.1038/s43705-022-00188-3>

12. Wood, Derrick. E., and Steven. L. Salzberg. 2014. "Kraken: ultrafast metagenomic sequence classification using exact alignments." *Genome Biology* 15(3): R46. <https://doi.org/10.1186/gb-2014-15-3-r46>

13. Bankevich, Anton., Sergey. Nurk, Dmitry. Antipov, Alexey. A. Gurevich, Mikhail. Dvorkin, Alexander. S. Kulikov, Valery. M. Lesin, et al. 2012. "SPAdes: a new genome assembly algorithm and its applications to single-cell sequencing." *Journal of Computational Biology* 19(5): 455-77. <https://doi.org/10.1089/cmb.2012.0021>

14. Laetsch, Dominik, and Mark Blaxter. 2017. "BlobTools: Interrogation of genome assemblies." *F1000Res* 6: 16. <https://doi.org/10.12688/f1000research.12232.1>

15. Seemann, Torsten. 2014. "Prokka: rapid prokaryotic genome annotation." *Bioinformatics* 30(14): 2068-9. <https://doi.org/10.1093/bioinformatics/btu153>

16. Parks, Donovan. H., Michael. Imelfort, Connor. T. Skennerton, Philip. Hugenholtz, and Gene. W. Tyson. 2015. "CheckM: assessing the quality of microbial genomes recovered from isolates, single cells, and metagenomes." *Genome Research* 25(7): 1043-55. <https://doi.org/10.1101/gr.186072.114>

17. Lee, Imchang., Yeong. Ouk Kim, Sang. Cheol. Park, and Jongsik. Chun. 2016. "OrthoANI: An improved algorithm and software for calculating average nucleotide identity." *International Journal of Systematic and Evolutionary Microbiology* 66(2): 1100-03. <https://doi.org/10.1099/ijsem.0.000760>

18. Treangen, Todd J., Brian D. Ondov, Sergey Koren, and Adam M. Phillippy. 2014. "The Harvest suite for rapid core-genome alignment and visualization of thousands of intraspecific microbial genomes." *Genome Biology* 15(11): 524. <https://doi.org/10.1186/s13059-014-0524-x>

19. Tamura, Koichiro, Glen Stecher, and Sudhir Kumar. 2021. "MEGA11: Molecular Evolutionary Genetics Analysis Version 11." *Molecular Biology and Evolution* 38(7): 3022-27. <https://doi.org/10.1093/molbev/msab120>

20. Xu, Shuangbin, Lin Li, Xiao Luo, Meijun Chen, Wenli Tang, Li Zhan, Zehan Dai, et al. 2022. "Ggtree: A serialized data object for visualization of a phylogenetic tree and annotation data." *iMeta* 1(4): e56. <https://doi.org/10.1002/imt2.56>
